# Real‐time social selection maintains honesty of a dynamic visual signal in cooperative fish

**DOI:** 10.1002/evl3.24

**Published:** 2017-10-25

**Authors:** Judith C. Bachmann, Fabio Cortesi, Matthew D. Hall, N. Justin Marshall, Walter Salzburger, Hugo F. Gante

**Affiliations:** ^1^ Zoological Institute, Vesalgasse 1 University of Basel 4051 Basel Switzerland; ^2^ Department of Evolutionary Biology and Environmental Studies University of Zurich 8057 Zurich Switzerland; ^3^ Queensland Brain Institute University of Queensland Brisbane Queensland 4072 Australia; ^4^ School of Biological Sciences Monash University Melbourne 3800 Australia

**Keywords:** Costly signaling theory, conventional signal, cichlid, *Neolamprologus*, out‐of‐equilibrium, pigmentation, receiver retaliation costs, reliability, strategic costs, visual model

## Abstract

Our understanding of animal communication has been largely driven by advances in theory since empirical evidence has been difficult to obtain. Costly signaling theory became the dominant paradigm explaining the evolution of honest signals, according to which communication reliability relies on differential costs imposed on signalers to distinguish animals of different quality. On the other hand, mathematical models disagree on the source of costs at the communication equilibrium. Here, we present an empirical framework to study the evolution of honest signals that generates predictions on the form, function, and sources of reliability of visual signals. We test these predictions on the facial color patterns of the cooperatively breeding Princess of Burundi cichlid, *Neolamprologus brichardi*. Using theoretical visual models and behavioral experiments we show that these patterns possess stable chromatic properties for efficient transmission in the aquatic environment, while dynamic changes in signal luminance are used by the fish to communicate switches in aggressive intent. By manipulating signal into out‐of‐equilibrium expression and simulating a cheater invasion, we demonstrate that social costs (receiver retaliation) promote the honesty of this dynamic conventional signal. By directly probing the sender of a signal in real time, social selection is likely to be the mechanism of choice shaping the evolution of inexpensive, yet reliable context‐dependent social signals in general.

**Impact Summary**

The principles guiding animal communication and the evolution of animal signals have long inspired researchers. What prevents animals from lying, communication from losing meaning and the signaling systems from collapsing? Theoreticians concluded that animal signals need costs to be made reliable, although there is disagreement regarding the source and type of those costs. Experimental data could shed light on the evolution of animal signals and communication, although attempts to determine the exact source of reliability costs have had limited success. In this study, we devised a framework that aids the empirical study of the evolution of honest communication by examining the form, function, and sources of reliability of visual signals. We show the usefulness of this framework by examining the facial mask of the cooperative Princess of Burundi cichlid fish and uncovering how fish reliably communicate aggressiveness and dominance. We found that much like the colorful masks used by humans in the Mexican‐free wrestling Lucha Libre, the facial mask of these fish is highly conspicuous, the colors encode the fish's intentions to fight and the fish must reliably display those colors—if they cheat, they are punished. Thus, we show that reliability of animal communication can be maintained by social policing and does not require additional costs.

How honest signals evolve is hotly debated by animal communication theoreticians (Grose 2011). An appealing solution to the puzzling problem of signaling honesty when signaler and receiver interests do not coincide lies in the existence of costs (Bradbury and Vehrencamp 2011). According to costly signaling theory, honesty of a message is maintained by differential costs of signaling imposed on animals of different quality (Grafen 1990; Bradbury and Vehrencamp 2011; Fraser 2012; Higham 2014). Nevertheless, there is considerable disagreement regarding the exact nature of these costs. On one side, the handicap principle requires costs paid by all signalers at the equilibrium (Zahavi 1975; John Maynard‐Smith and Harper 2003; Hurd and Enquist 2005; Searcy and Nowicki 2005). In spite of the generalized acceptance of handicaps, reinterpretation of earlier models and new mathematical simulations all conclude that strategic costs at the communication equilibrium are not sufficient, nor even necessary, for reliable signaling, a conclusion that is nonetheless still not widely recognized by empirical biologists testing honesty in communication (e.g., Hurd 1997; Getty 1998a, 1998b, 2006; Lachmann et al. 2001; Számadó 2011a). Instead, honesty of signaling systems can be socially selected and context‐dependent, such that social selection would act at the individual level during competition for nonreproductive resources (Tanaka 1996). Rather than incurring realized strategic costs at every signaling event, potential social costs imposed by receivers on dishonest signalers could explain how some types of cheap honest signals evolve. Such is the case of conventional signals, which would not need any realized strategic costs on top of the efficacy costs that signal transmission entails (Hurd 1995; Tanaka 1996; Lachmann et al. 2001).

To evaluate alternative scenarios for the evolution of honest signals it is necessary to empirically measure marginal costs of cheating in manipulated out‐of‐equilibrium signals, where individuals are forced to exhibit unreliable signal expression (Kotiaho 2001; Lachmann et al. 2001; Számadó 2011b, 2012; Higham 2014). While the understanding of honest signaling in animal communication has centered around questions related to the origin of costs of communicating, some authors have questioned whether signaling costs have even been determined empirically (e.g., Fraser 2012; Számadó 2012; Higham 2014). Here, we combine conceptual approaches from visual modeling (Vorobyev and Osorio 1998; Vorobyev et al. 2001) and signaling theory (e.g., Laidre and Johnstone 2013) into a 3‐stage framework that generates testable predictions about the evolution of form, function, and sources of reliability of color signals, making the demonstration of the existence of strategic costs a more tractable empirical problem (Fig. 1). We follow Higham's (Higham 2014) definition of costly signaling and Fraser's (Fraser 2012) classification of signal costs. We allow cost functions to be zero at the equilibrium, to include social selection through receiver punishment as a mechanism that can generate marginal costs to cheaters and maintain signaling reliability (as elaborated elsewhere (Fraser 2012)). Furthermore, recent mathematical models indicate that the evolution of index signals can also be explained by differential costs (Biernaskie et al. 2014). Therefore, both handicaps and indices share a link to *intrinsic* physiological condition to guarantee honesty, while conventional signals rely on *extrinsic* sources of reliability. Whether liar detection mechanisms have evolved helps determine if intrinsic constraints or extrinsic socially imposed costs exist: liar detection is expected to evolve only in cheap conventional signals, where receivers can immediately probe senders (i.e., in real time). Conversely, in intrinsically costly handicaps or indices, reliability is verified in terms of viability and fecundity, too far into the future for social selection to be effective (Lachmann et al. 2001).

**Figure 1:**
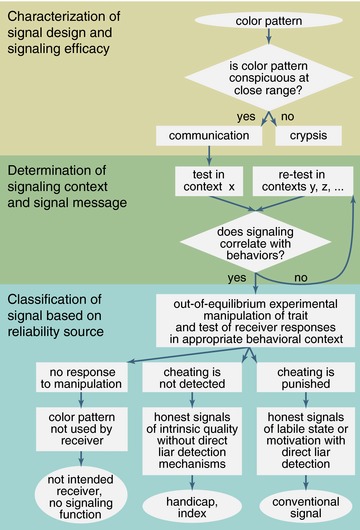
A framework for studying intraspecific color signals. Flowchart of the 3‐stage framework proposed for studying intraspecific color signals generates predictions to determine signal efficacy, function, and proximate reliability mechanisms.

Here, we implement this framework to study the evolution of visual signals in the facial mask of the Princess of Burundi cichlid, *Neolamprologus brichardi* (Fig. 2), as we observed fast and transitory changes to the intensity of the black horizontal stripe during agonistic social interactions. Accordingly, we first calculated visual models to quantify signal design and signaling efficacy, and to identify the axis of variation for experimental manipulation. We then used behavioral experiments to determine the message conveyed by our visual signal of interest. Finally, by experimentally manipulating sender signals into an out‐of‐equilibrium state and recording receiver's reactions, we identified the class of costs that unreliable signaling incurs.

**Figure 2:**
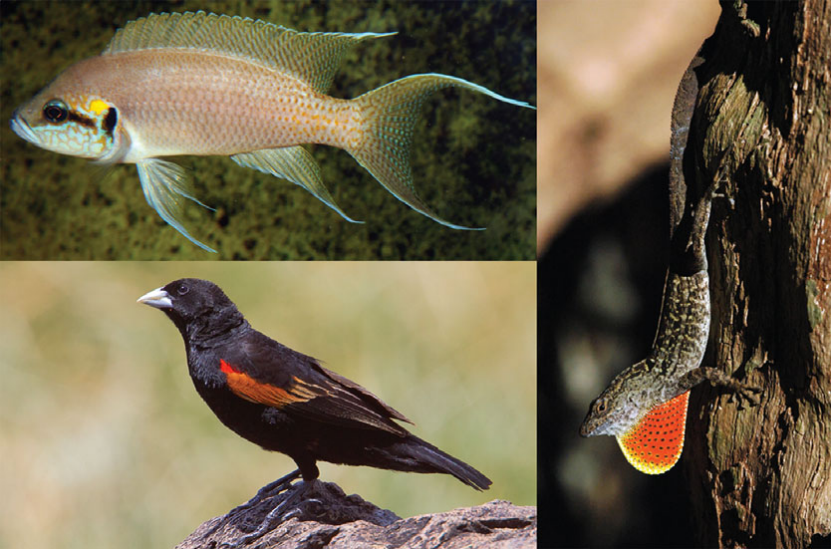
Dynamic visual signals of territoriality and aggressive intent. Territorial species display a variety of conspicuous visual signals to communicate aggressive intent. To decrease predation pressure and in nonaggressive contexts several species use morphological, physiological, or behavioral adaptations to conceal signals. We show that rapid physiological color changes, achieved by pigment movement in melanophores (black pigment cells), are a cheap proximate mechanism turning a visual signal of aggressive intent “on” or “off” in lifelong territorial fish. Clockwise from top left: black horizontal stripe in dominant Princess of Burundi cichlid *(Neolamprologus brichardi)*; extended dewlap in trunk‐ground Brown Anole *(Anolis sagrei)* (Losos 2009); partially covered epaulette in Fan‐tailed Widowbird *(Euplectes axillaris)* (Pryke and Andersson 2003).

## Methods

### NEOLAMPROLOGUS BRICHARDI

Princess cichlids emerged as prime model systems for studying the evolution of cooperative breeding behavior (Wong and Balshine 2011), and substantial genomic and transcriptomic resources are available (Brawand et al. 2014; Gante et al. 2016). Like most other species of the Tanganyikan cichlid tribe Lamprologini, *N. brichardi* is sexually monochromatic, that is coloration patterns are identical between males and females (Gante and Salzburger 2012). The dominant, breeding couple is aided by up to 25 subordinate helpers, and the social group is organized in a strict linear hierarchy (Balshine et al. 2001; Zöttl et al. 2013). Conflict and aggression levels are highest between individuals of the same sex and similar size (Mitchell et al. 2009; Wong and Balshine 2011; Garvy et al. 2015). As a consequence of cooperative breeding and colony life, Princess cichlids repeatedly and regularly interact and communication between group members, mates, and neighbors likely involves multiple signal modalities, such as olfactory, visual, and auditory (Balshine‐Earn and Lotem 1998; Frostman and Sherman 2004; Le Vin et al. 2010; Kohda et al. 2015; Spinks et al. 2017). Further details on biology and husbandry in Supplementary File.

### STAGE 1—CHARACTERIZATION OF SIGNAL DESIGN AND SIGNALING EFFICACY

We used a theoretical visual model (Vorobyev and Osorio 1998; Vorobyev et al. 2001) to quantify the chromatic and achromatic contrasts between the facial pattern elements using the *N. brichardi* visual system under ambient light conditions from their natural habitat (further details in Supplementary File; Figs. S1 and S2). To test whether the facial color pattern is conspicuous to the fish eye, we compared chromatic and achromatic contrasts between adjacent and nonadjacent color patches of dominant fish (i.e., fish with dark horizontal stripes, which is the state in which the phenotype is normally expressed). High visual conspicuousness is achieved by stimulation of adjacent photoreceptors in opposite ways by complementary radiance spectra (Lythgoe 1979; Hurvich 1981). Thus, signal design strategies for increased conspicuousness and transmission efficacy include the use of (*i*) white or highly reflective colors adjacent to dark patches, (*ii*) adjacent patches with complementary colors and (*iii*) color combinations centered or just offset the transmission maxima of the media, in this case the underwater habitats where the species evolved (Lythgoe 1979; 1992). Further, (*iv*) a visual signal in a particular light environment is most conspicuous when adjacent color elements have greater contrasts than nonadjacent elements (Endler 1992, 2012; Guilford and Dawkins 1993). To detect overall differences between adjacent and nonadjacent color patches in chromatic and achromatic contrasts in dominant fish, we ran linear mixed models (LMMs) in R package nlme (Pinheiro et al. 2017) with contrasts as responses, adjacency as explanatory variable and fish as random effect.

### STAGE 2—DETERMINATION OF SIGNALING CONTEXT AND SIGNAL MESSAGE

Given the higher potential conflict between individuals of the same sex and similar size, territorial combats were staged such that both fish have simultaneous ownership over a territory and that they cannot divide this resource after the barrier is removed (further details in Supplementary File; Fig. S3). Twenty fish dyads were matched by sex, standard length (mean difference: 0.2 cm ± 0.45 (s.d.); Mann–Whitney U test, V = 233, *P* = 0.27) and body mass (mean difference: 0.78 g ± 1.25 (s.d.); Mann–Whitney U test, V = 316, *P* = 0.21).

Intensity of the horizontal facial stripe was categorized by eye (pale or dark) at the beginning and at the end of experiments. Combat success (winning or losing) and behaviors of the 20 min combats were video recorded with a Sony HDR XR 550VE. A fighting ability index for each fish was calculated as the difference between aggressive and submissive behaviors (Table S1; dominance index in (Aubin‐Horth et al. 2007)).

To determine whether body mass and fighting ability differed between winners and losers, and if it was the same for males and females, we used LMMs, with body mass and fighting ability as responses and combat success, sex, and their interaction as explanatory variables, as well as pair as random effect. To test whether combat success is associated to the intensity of the facial stripe at the beginning or at the end of the contest we fitted generalized linear‐mixed models (GLMMs) with binomial error distribution, logit link function, and pair as random effect in R package lme4 (Bates et al. 2015).

We calculated Mann–Whitney U tests applying false discovery rate to determine which color elements change in chromatic or achromatic contrasts with switches in dominance (darkening or paling of the horizontal stripe). To detect overall differences between adjacent and nonadjacent color patches in chromatic and achromatic contrasts, in dominant and subordinate fish, we ran two LMMs with contrasts as responses and adjacency, stripe intensity, and their interaction as explanatory variables. As we measured several color patches per fish and then used them in different comparisons, all adjacent and all nonadjacent chromatic or achromatic contrasts were averaged per individual. Individual was used as random effect. Shapiro tests confirmed normality of chromatic contrast and square‐rooted achromatic contrast residuals.

### STAGE 3—CLASSIFICATION OF SIGNAL BASED ON RELIABILITY SOURCE

To empirically measure marginal costs of cheating and identify the class of signaling costs, we manipulated horizontal facial stripes into two extreme out‐of‐equilibrium states, where individuals were forced to exhibit unreliable signal expression. Each fish was tested twice with two of the following three treatments in randomized order: 1. Darkened facial stripe; 2. Paled facial stripe; and 3. Control sham‐manipulation (further details in Supplementary File). Spectral reflectance measurements confirm that both out‐of‐equilibrium treatments resulted in the desired effect of extreme darkening and paling along the axis of normal variation, such that an enhanced stripe is darker, while the subdued stripe is paler, than all control nonmanipulated stripes (Fig. S4; Table S2). By testing multiple manipulations on each individual we can control for aggression biases due to individual differences such as personality (Bell 2007).

We used standard mirror image stimulation (MIS) to determine if *N. brichardi* are able to detect and punish unreliable signals by measuring the response of each individual to its own reflection. Cichlids, including *Neolamprologus*, are known to react aggressively toward their mirror images (Balzarini et al. 2014), therefore MIS provides instantaneous feedback without some of the confounding factors resulting from using other individuals as stimuli (Rowland 1999). In our setup, the focal fish act as intruders in territories of individuals of the same size they perceive as territory owners that show the manipulated or control signals (Fig. S5). This addresses common limitations of studies that present manipulated individuals to focal dominant, territorial individuals, which end up testing the response of territory holders to intruders displaying different signals, rather than testing the repellent effect of a signal displayed by territorial individuals (Bradbury and Vehrencamp 2011). Therefore, we can focus on reactions toward the manipulated stripe alone, including testing the behavior of nonterritorial individuals, which are the ones most interested in detecting unreliable signals if used by territory‐holding individuals. Testing nonterritorial individuals removes motivational differences between individuals (generated by the value given to a territorial resource by territorial fish) and testing only one individual against itself removes resource holding potential differences (generated by body size or condition differences whenever two individuals interact).

We fit two different LMMs with aggressive bouts or latency to attack as response variables and treatment, sex and their interaction as explanatory variables. In the case of aggressive bouts residuals were normalized using square‐root transformation. As fish were tested twice, individual and treatment order were added as random effects. Tukey's HSD post‐hoc analysis was performed to test for differences among treatment levels.

In our behavioral assay to determine sources of reliability for signal classification, (*i*) if stripe intensity does not encode individual fighting abilities (but simply correlates with them) or if the intended receiver is other than the one tested, we do not expect to observe a response to the manipulation (i.e., no differences in aggression toward manipulated or nonmanipulated individuals). On the other hand, (*ii*) if stripe intensity signals a context‐independent (i.e., intrinsic) quality whereby realized strategic costs guarantee honesty (in the case of a handicap or index), receivers should not challenge individuals that signal dominance (even in the presence of cheaters with enhanced signals) but should do so toward subordinate individuals (including those with artificially subdued signals). In this case, we expect higher aggression levels, lower latency to attack, or both with decreasing signal intensity (from enhanced, to control, to subdued). This would constitute an innate fear response whereby higher stimulus intensity would evoke larger visual receptor potentials that would cause both a greater number and higher frequency of action potentials in the fear centers of the brain (Martin 1991). Alternatively, (*iii*) if stripe intensity signals context‐dependent dominance whereby social selection (i.e., detection and punishment of cheaters) maintains signal honesty, we expect increased levels of aggression toward an unreliable signal (i.e., a conventional signal). In the case of long‐term commitment to defend a resource, we expect higher aggression rates, lower latency to attack, or both toward senders of unreliable signals (both enhanced and subdued), otherwise it is possible that only enhanced signals will be detected and punished (Owens and Hartley 1991; Számadó 2011a). This would constitute a learned behavior since response intensity and latency would not correlate with stimulus intensity (Dawkins and Guilford 1991).

## Results and Discussion

### STAGE 1 – CHARACTERIZATION OF SIGNAL DESIGN AND SIGNALING EFFICACY: HIGH CHROMATIC CONSPICUOUSNESS OF *N. BRICHARDI*’S FACIAL COLORATION

**Figure 3:**
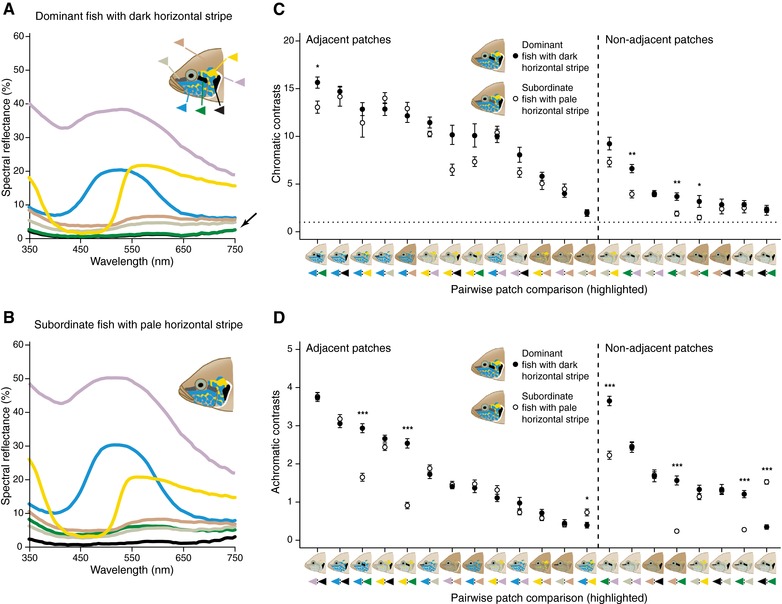
Color properties of facial elements in dominant and subordinate *Neolamprologus brichardi*. (A and B) Mean spectral reflectance of facial color patches. (A) Horizontal (green triangle) and vertical (black triangle) facial stripes have the same reflectance in dominant fish (note arrow). (B) Losing a combat and becoming subordinate significantly increases reflectance of horizontal facial stripe in subordinate fish, that is paling occurs. (C and D) Chromatic and achromatic contrasts (mean ± SEM) between pairs of adjacent and nonadjacent color patches as perceived by *N. brichardi*, ordered from highest to lowest in dominant fish. (C) High chromatic contrast Δ*S* is achieved by any combination of blue, yellow, and black patches. Stippled line marks the 1 JND (just noticeable difference), threshold after which two colors are thought to be perceived as different. (D) High achromatic contrast Δ*L* is achieved by combining black melanic stripes and other patches. Asterisks illustrate significant differences in contrast between dominant and subordinate fish (^***^ *P* < 0.001, ^**^ *P* < 0.01, ^*^ *P* < 0.05).

Unambiguous communication selects for signals that promote effective stimulation of sensory systems relative to environmental noise and signal degradation. Using spectral reflectance measurements and theoretical fish visual models we show that the facial color pattern in dominant *N. brichardi* achieves high chromatic conspicuousness to the visual system of conspecifics (Fig. 3A and C, filled circles). This signal design is exceptionally effective and ensures transmission efficacy in the aquatic environment: (*i*) white is a broadband optical reflector, reflecting across all the available light spectrum and the structural blue patches reflect the high‐intensity wavelengths available underwater, while (*ii*) the adjacent black melanic stripes absorb most incident light. Chromatic contrast is further achieved by (*iii*) the use of complementary colors, blue and yellow, centered in the highest light intensity of water transmission. Finally, (*iv*) chromatic contrasts differ between adjacent and nonadjacent patches (linear mixed‐effects model [LMM]: *F*_1,9_ = 207.31, *P* < 0.001) and all pairwise color comparisons are well above the just noticeable difference (JND) threshold of one, a standard in chromatic color discrimination (Vorobyev and Osorio 1998; Endler 2012) (Fig. 3C, filled circles). Compared to chromatic contrasts, the overall variance in achromatic contrasts is smaller, and adjacent and nonadjacent elements do not greatly differ from one another (LMM: *F*_1,9_ = 4.61, *P* = 0.06; Fig. 3D, filled circles).

### STAGE 2 – DETERMINATION OF SIGNALING CONTEXT AND SIGNAL MESSAGE: *NEOLAMPROLOGUS BRICHARDI* MAKE DYNAMIC AND CONTEXT‐DEPENDENT USE OF FACIAL SIGNAL

**Figure 4:**
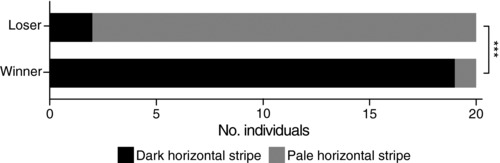
Horizontal facial stripe provides information on aggressive intent. Facial stripe intensity is associated with fighting ability and dominance (winning or losing) at the end of a combat. L: losers; W: winners. Winners show a dark horizontal stripe, while losers pale theirs (^***^ *P* < 0.001).

High chromatic conspicuousness of the facial pattern implicates selection for unambiguous signaling, at least at close range (Fig. 1). We thus tested the likely function in communication of different elements of the facial pattern, by staging dyadic combats of territory‐holding fish. As expected, body size (LMM: *F*_1,18_ = 8.79, *P* = 0.01) and fighting ability (LMM: *F*_1,18_ = 32.11, *P* < 0.001) differed between winners and losers of staged combats, irrespective of sex (LMM: *F*_1,18_ = 1.85, *P* = 0.19 and LMM: *F*_1,18_ = 0.04, *P* = 0.85) or their interaction (LMM: *F*_1,18_ = 0.44, *P* = 0.52 and LMM: *F*_1,18_ = 0.58, *P* = 0.46; Fig. S8). Most importantly, we found that a change in aggressive intent by losers of the combat leads to a rapid paling of the horizontal facial stripe at the end of the contest (generalized linear mixed‐effects model [GLMM] with binomial error distribution: $\chi_{1}^{2}$ = 14.97, *P* < 0.001; Figs. 3B, 4, S6, S7, S9). Hence changes in horizontal stripe intensity dynamically reflect an individual's instantaneous motivation to fight, its aggressive intent and current dominance, while not predicting future contest outcome (binomial GLMM: $\chi_{1}^{2}$ = 0.01, *P* = 0.93) nor aggression level (LMM: *F*_1,17_ = 0.72, *P* = 0.41). These results indicate that this signal is fundamentally different from other well‐described signals that function as badges of status, which by definition predict the outcome of future contests and provide more stable rank information (Tibbetts and Dale 2004; Bradbury and Vehrencamp 2011). Such paling or darkening achieved by rapid movement of pigments within melanophores (black pigment cells) is a cheap physiological response available to many lower vertebrates (e.g., fish, reptiles) and invertebrates (e.g., cephalopods), and can occur within a few seconds in fish (Muske and Fernald 1987; Fujii 2000).

Next, we used theoretical visual models to test whether the observed physiological paling of the horizontal stripe induces changes in conspicuousness of the overall facial pattern. We found that *chromatic* conspicuousness is unaffected even after paling takes place (Figs. 3C and S10A). In particular, high *chromatic* contrast is still achieved by higher contrast of adjacent patches than nonadjacent patches (LMM: *F*_1,18_ = 208.21, *P* < 0.001) and not by differences in stripe darkness (LMM: *F*_1,18_ = 3.48, *P* = 0.08) or interaction between adjacency and stripe darkness (LMM: *F*_1,18_ = 0.05, *P* = 0.82). This model explains 99.31% of chromatic contrast variance, 96.50% of which is explained by adjacency of the color elements, while changes in luminance of the horizontal stripe explain the remaining variance. On the other hand, we found that *achromatic* contrasts become more relevant with changes in luminance of the horizontal stripe (LMM: *F*_1,18_ = 9.11, *P* = 0.007), as adjacent contrasts are higher than nonadjacent contrasts (LMM: *F*_1,18_ = 5.07, *P* = 0.037) and this difference is larger in submissive fish (LMM: *F*_1,18_ = 6.78, *P* = 0.018; Figs. 3D and S10B). This model explains 95.90% of the achromatic contrast variance, 68.53% of which is explained by changes in darkness of the horizontal stripe, 22.34% by signal design (patch adjacency) and the remainder 5.02% by their interaction. Thus, we find that compared to the horizontal black stripe the white, yellow, and blue are less dynamic elements of the facial mask in *N. brichardi*, and seem to provide little or no information regarding changes in aggressive intent. Instead they act as amplifiers to enhance pattern conspicuousness and changes in luminance of the horizontal stripe. Using this dual mechanism is an elegant way to ensure that conspicuousness, and hence communication efficacy, does not decrease due to context‐dependent signaling.

### STAGE 3 – CLASSIFICATION OF SIGNAL BASED ON RELIABILITY SOURCE: SOCIAL SELECTION IS THE PROXIMATE MECHANISM PRODUCING AN EVOLUTIONARY STABLE SIGNALING STRATEGY

**Figure 5:**
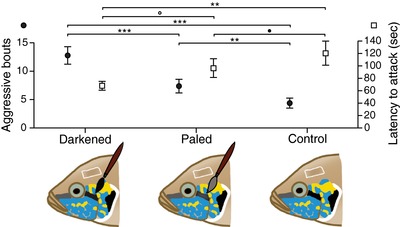
Social selection maintains reliable communication. Social costs (aggressive bouts, filled squares; latency to attack, empty squares) on out‐of‐equilibrium and control signals. Unreliable signaling of strength (darkened stripe) and weakness (paled stripe) is punished by increased and faster costs relative to reliable signaling (control). Mean ± SEM are shown. Symbols illustrate significant differences in pairwise post‐hoc tests between treatments (^***^ *P* < 0.001, ^**^ *P* < 0.01, • *P* < 0.1, ◯ *P* > 0.1).

To identify the class of costs maintaining honest communication we manipulated the luminance of the horizontal facial stripe into out‐of‐equilibrium states. We found that receivers actively “read” and quickly react to the manipulations, detecting and punishing cheaters in real time (Figs. 5 and S11). Manipulation of the horizontal stripe had an effect on the number of aggressive bouts received (LMM: *F*_2,45_ = 13.73, *P* < 0.001), irrespective of sex (LMM: *F*_2,45_ = 0.48, *P* = 0.62). Individuals with darkened stripes received 1.6 times more aggression than individuals with paled stripes (Tukey HSD: *z* = –3.89, *P* < 0.001) and 2.6 times more than controls (Tukey HSD: *z* = –6.59, *P* < 0.001). Importantly, individuals with paled stripes received 1.5 times more aggression than controls (Tukey HSD: *z* = –2.97, *P* = 0.008), indicating that unreliable signaling brings increased marginal costs to all types of cheaters. We also found that unreliable signals induce shorter latencies to aggression (LMM: *F*_2,44_ = 7.19, *P* = 0.002). Although less clear‐cut than aggression level, individuals with artificially darkened and paled stripes received aggression with 1.7 and 1.2 times shorter latency than controls, respectively (Tukey HSD: *z* = 3.43, *P* = 0.002 and *z* = 2.07, *P* = 0.095, respectively; Figs. 5 and S11).

Physiological color changes have previously been implicated in signaling aggressive intent in a number of taxa, in particular fish (Muske and Fernald 1987; Korzan et al. 2002; Moretz and Morris 2003; Rodrigues et al. 2009). Higher levels of aggression toward the signal reported in some of these studies were interpreted as receiver retaliation costs maintaining honesty of a conventional signal, although it is not a sufficient condition. We show here that *unreliable* signaling has increased costs relative to reliable signaling, which is pivotal to the evolution of honest signals (Lachmann et al. 2001; Searcy and Nowicki 2005; Higham 2014). Our study provides rare empirical evidence that, similar to paper wasps (Tibbetts and Dale 2004; Tibbetts and Izzo 2010), fish are able to detect and punish individuals that signal unreliably, be they cheaters signaling strength (“bluffers”) or modest liars (“Trojans”). This ability is likely part of the social competence repertoire learned early in life by *Neolamprologus* (Taborsky and Oliveira 2012).

## Conclusions

Our framework for studying signal evolution proved important in generating testable predictions emerging from theory. We demonstrated that the facial mask of *N. brichardi* has stable chromatic properties that keep signaling efficacy high at all times, while rapid physiological changes in luminance of just one element (the horizontal melanic stripe) dynamically communicate reversals in aggressive intent and dominance. We further demonstrated that real‐time social selection maintains honesty of the signaling system. Thus, as with communication efficacy, we demonstrate that communication reliability does not decrease due to context‐dependent signaling but is rather promoted by social policing in *Neolamprologus*. Since aggressive intent is not easily handicapped (Bradbury and Vehrencamp 2011), receivers can directly assess reliability of signals of aggressive intent with relative ease (Lachmann et al. 2001) and impose social costs on cheaters, promoting the evolution of signal honesty. We thus provide empirical support to theoretical models concluding that honest communication does not require differential strategic costs and that reliability can indeed be guaranteed by mechanisms that promote low realized costs for honest signalers, such as through social selection (Hurd 1995; Tanaka 1996; Lachmann et al. 2001; Számadó 2011b). Since receivers can effectively probe reliability of signals in real time (essentially instantaneously), we propose that social selection and cheap conventional signals are expected to be a widely chosen solution for honest context‐dependent, social signaling. Moreover, the design of social signals should follow efficacy considerations to be easily detectable (high signal‐to‐noise ratio), discriminable (have more or less discrete states), and memorable to receivers (a code that is easy to learn) (Guilford and Dawkins 1991), like the facial mask of *N. brichardi*.

Taken together, these findings suggest that social selection may contribute to the dramatic diversity of color patterns (stripes, bars, blotches) observed in many sexually monochromatic cichlids (Gante and Salzburger 2012) as another form of selection shaping diversity in this clade (Salzburger 2009; Muschick et al. 2012). Social selection is expected to drive rapid signal evolution especially in isolated allopatric populations (West‐Eberhard 1983; Tanaka 1996). While we expect socially selected signals to be sexually monomorphic because of similar selection regimes between sexes, most research into color signaling in cichlids has centered on sexually dichromatic traits (Maan and Sefc 2013), in particular in assemblages from Lake Malawi and Lake Victoria (Wagner et al. 2012). Our results point to rapid social trait evolution as another process potentially affecting speciation dynamics in cichlids. Confirmation of its importance would raise social selection to the level of sexual and natural selection in shaping adaptive radiations of cichlid fishes.

Associate Editor: A. Charmantier

## Supporting information


**Figure S1**. Data on the visual system of *Neolamprologus brichardi* and its light environment used to build visual models.
**Figure S2**. Data on the light environment of *Neolamprologus brichardi* used to build visual models.
**Figure S3**. Schematic representation of resource contest setup.
**Figure S4**. Out‐of‐equilibrium manipulation of the horizontal facial stripe along axis of normal trait variation.
**Figure S5**. Schematic representation of standard mirror image experimental setup.
**Figure S6**. Spectral properties of dominant and subordinate individuals.
**Figure S7**. Maxwell color triangles of dominant and subordinate individuals.
**Figure S8**. Resource contest experiment.
**Figure S9**. Aspect of subordinate individual.
**Figure S10**. Spectral properties of dominant and subordinate individuals and impacts of paling on contrasts.
**Figure S11**. Aggression level and latency to aggression induced by out‐of‐equilibrium manipulations of the horizontal facial stripe.
**Table S1**. Ethogram of behavior repertoire of *Neolamprologus brichardi*
**Table S2**. Cluster analysis of principal components of spectral data

## References

[evl324-bib-0001] Aubin‐Horth, N. , J. K. Desjardins , Y. M. Martei , S. Balshine , and H. A. Hofmann . 2007 Masculinized dominant females in a cooperatively breeding species. Mol. Ecol. 16:1349–1358. 17391260 10.1111/j.1365-294X.2007.03249.x

[evl324-bib-0002] Balshine, S. , B. Leach , F. Neat , H. Reid , M. Taborsky , and N. Werner . 2001 Correlates of group size in a cooperatively breeding cichlid fish (*Neolamprologus pulcher*). Behav. Ecol. Sociobiol. 50:134–140.

[evl324-bib-0003] Balshine‐Earn, S. , and A. Lotem . 1998 Individual recognition in a cooperatively breeding cichlid: evidence from video playback experiments. Behaviour 135:369–386.

[evl324-bib-0004] Balzarini, V. , M. Taborsky , S. Wanner , F. Koch , and J. G. Frommen . 2014 Mirror, mirror on the wall: the predictive value of mirror tests for measuring aggression in fish. Behav. Ecol. Sociobiol. 68:871–878.

[evl324-bib-0005] Bates, D. , M. Maechler , B. Bolker , and S. Walker . 2015 lme4: linear mixed‐effects models using eigen and S4. J. Stat. Softw. 67:1–48.

[evl324-bib-0006] Bell, A. M. 2007 Future directions in behavioural syndromes research. Proc. R. Soc. B Biol. Sci. 274:755–761. 10.1098/rspb.2006.0199 PMC1919401 17251088

[evl324-bib-0007] Biernaskie, J. M. , A. Grafen , and J. C. Perry . 2014 The evolution of index signals to avoid the cost of dishonesty. Proc. R. Soc. B Biol. Sci. 281:20140876. 10.1098/rspb.2014.0876 PMC4123701 25056623

[evl324-bib-0008] Bradbury, J. W. , and S. L. Vehrencamp . 2011 Principles of animal communication, 2nd ed Sinauer Associates, Inc, Sunderland, MA.

[evl324-bib-0009] Brawand, D. , C. E. Wagner , Y. I. Li , M. Malinsky , I. Keller , S. Fan , O. Simakov , A. Y. Ng , Z. W. Lim , E. Bezault , et al. 2014 The genomic substrate for adaptive radiation in African cichlid fish. Nature 513:375–381. 25186727 10.1038/nature13726 PMC4353498

[evl324-bib-0010] Dawkins, M. S. , and T. Guilford . 1991 The corruption of honest signalling. Anim. Behav. 41:865–873.

[evl324-bib-0011] Endler, J. A. 1992 Signals, signal conditions, and the direction of evolution. Am. Nat. 139:S125–S153.

[evl324-bib-0012] Endler, J. A. 2012 A framework for analysing colour pattern geometry: adjacent colours. Biol. J. Linn. Soc. 107:233–253.

[evl324-bib-0013] Fraser, B. 2012 Costly signalling theories: beyond the handicap principle. Biol. Philos. 27:263–278.

[evl324-bib-0014] Frostman, P. , and P. T. Sherman . 2004 Behavioral response to familiar and unfamiliar neighbors in a territorial cichlid, Neolamprologus pulcher. Ichthyol. Res. 51:8–10.

[evl324-bib-0015] Fujii, R. 2000 The regulation of motile activity in fish chromatophores. Pigment Cell Res. 13:300–319. 11041206 10.1034/j.1600-0749.2000.130502.x

[evl324-bib-0016] Gante, H. F. , M. Matschiner , M. Malmstrøm , K. S. Jakobsen , S. Jentoft , and W. Salzburger . 2016 Genomics of speciation and introgression in Princess cichlid fishes from Lake Tanganyika. Mol. Ecol. 25:6143–6161. 27452499 10.1111/mec.13767

[evl324-bib-0017] Gante, H. F. , and W. Salzburger . 2012 Evolution: cichlid models on the runaway to speciation. Curr. Biol. 22:R956–R958. 23174298 10.1016/j.cub.2012.09.045

[evl324-bib-0018] Garvy, K. A. , J. K. Hellmann , I. Y. Ligocki , A. R. Reddon , S. E. Marsh‐Rollo , I. M. Hamilton , S. Balshine , and C. M. O'Connor . 2015 Sex and social status affect territorial defence in a cooperatively breeding cichlid fish, *Neolamprologus savoryi* . Hydrobiologia 748:75–85.

[evl324-bib-0019] Getty, T. 1998a Handicap signalling: when fecundity and viability do not add up. Anim. Behav. 56:127–130. 9710469 10.1006/anbe.1998.0744

[evl324-bib-0020] Getty, T. 1998b Reliable signalling need not be a handicap. Anim. Behav. 56:253–255. 9710484 10.1006/anbe.1998.0748

[evl324-bib-0021] Getty, T. 2006 Sexually selected signals are not similar to sports handicaps. Trends Ecol. Evol. 21:83–88. 16701479 10.1016/j.tree.2005.10.016

[evl324-bib-0022] Grafen, A. 1990 Biological signals as handicaps. J. Theor. Biol. 144:517–546. 2402153 10.1016/s0022-5193(05)80088-8

[evl324-bib-0023] Grose, J. 2011 Modelling and the fall and rise of the handicap principle. Biol. Philos. 26:677–696.

[evl324-bib-0024] Guilford, T. , and M. S. Dawkins . 1991 Receiver psychology and the evolution of animal signals. Anim. Behav. 42:1–14.

[evl324-bib-0025] Guilford, T. , and M. S. Dawkins . 1993 Receiver psychology and the design of animal signals. Trends Neurosci. 16:430–436. 7507611 10.1016/0166-2236(93)90068-w

[evl324-bib-0026] Higham, J. P. 2014 How does honest costly signaling work? Behav. Ecol. 25:8–11.

[evl324-bib-0027] Hurd, P. L. 1995 Communication in discrete action‐response games. J. Theor. Biol. 174:217–222.

[evl324-bib-0028] Hurd, P. L. 1997 Is signalling of fighting ability costlier for weaker individuals? J. Theor. Biol. 184:83–88.

[evl324-bib-0029] Hurd, P. L. , and M. Enquist . 2005 A strategic taxonomy of biological communication. Anim. Behav. 70:1155–1170.

[evl324-bib-0030] Hurvich, L. M. 1981 Colour vision (Sinauer Associates Inc., U.S.).

[evl324-bib-0031] Kohda, M. , L. A. Jordan , T. Hotta , N. Kosaka , K. Karino , H. Tanaka , M. Taniyama , and T. Takeyama . 2015 Facial recognition in a group‐living cichlid fish. PLoS One 10:1–12. 10.1371/journal.pone.0142552 PMC4659603 26605789

[evl324-bib-0032] Korzan, W. , T. Summers , and C. Summers . 2002 Manipulation of visual sympathetic sign stimulus modifies social status and plasma catecholamines. Gen. Comp. Endocrinol. 128:153–161. 12392689 10.1016/s0016-6480(02)00077-1

[evl324-bib-0033] Kotiaho, J. S. 2001 Costs of sexual traits: a mismatch between theoretical considerations and empirical evidence. Biol. Rev. Camb. Philos. Soc. 76:365–376. 11569789 10.1017/s1464793101005711

[evl324-bib-0034] Lachmann, M. , S. Számadó , and C. T. Bergstrom . 2001 Cost and conflict in animal signals and human language. Proc. Natl. Acad. Sci. USA 98:13189–13194. 11687618 10.1073/pnas.231216498 PMC60846

[evl324-bib-0035] Laidre, M. E. , and R. A. Johnstone . 2013 Animal signals. Curr. Biol. 23:R829–R833. 24070440 10.1016/j.cub.2013.07.070

[evl324-bib-0036] Losos, J. B. 2009 Lizards in an evolutionary tree: ecology and adaptive radiation of anoles. California Univ. Press, Berkeley.

[evl324-bib-0037] Lythgoe, J. N. 1979 The ecology of vision. Clarendon Press, Oxford.

[evl324-bib-0038] Maan, M. E. , and K. M. Sefc . 2013 Colour variation in cichlid fish: developmental mechanisms, selective pressures and evolutionary consequences. Semin. Cell Dev. Biol. 24:516–528. 23665150 10.1016/j.semcdb.2013.05.003 PMC3778878

[evl324-bib-0039] Martin, J. H. 1991 Coding and processing of sensory information Pp. 329–340 *in* Kandel E. R., Schwartz J. H., and Jessell T. M., eds. Principles of neural science. Elsevier Science Publishing Co, Inc., New York.

[evl324-bib-0040] Maynard‐Smith, J. , and D. Harper . 2003 Animal signals. Oxford Univ. Press, Oxford.

[evl324-bib-0041] Mitchell, J. , E. Jutzeler , D. Heg , and M. Taborsky . 2009 Dominant members of cooperatively‐breeding groups adjust their behaviour in response to the sexes of their subordinates. Behaviour 146:1665–1686.

[evl324-bib-0042] Moretz, J. A. , and M. R. Morris . 2003 Evolutionarily labile responses to a signal of aggressive intent. Proc. R. Soc. B Biol. Sci. 270:2271–2277. 10.1098/rspb.2003.2510 PMC1691502 14613614

[evl324-bib-0043] Muschick, M. , A. Indermaur , and W. Salzburger . 2012 Convergent evolution within an adaptive radiation of cichlid fishes. Curr. Biol. 22:2362–2368. 23159601 10.1016/j.cub.2012.10.048

[evl324-bib-0044] Muske, L. E. , and R. D. Fernald . 1987 Control of a teleost social signal. I. Neural basis for differential expression of a color pattern. J. Comp. Physiol. A 160:89–97. 3820134 10.1007/BF00613444

[evl324-bib-0045] Owens, I. P. F. , and I. R. Hartley . 1991 “Trojan Sparrows”: evolutionary consequences of dishonest invasion for the badges‐of‐status model. Am. Nat. 138:1187–1205.

[evl324-bib-0046] Pinheiro, J. , Bates, D. , DebRoy, S. , Sarkar, D. , and R Core Team . 2017 nlme: linear and nonlinear mixed effects models. R package version 3.1–131.

[evl324-bib-0047] Pryke, S. R. , and S. Andersson . 2003 Carotenoid‐based status signalling in red‐shouldered widowbirds (*Euplectes axillaris*): epaulet size and redness affect captive and territorial competition. Behav. Ecol. Sociobiol. 53:393–401.

[evl324-bib-0048] Rodrigues, R. R. , L. N. Carvalho , J. Zuanon , and K. Del‐Claro . 2009 Color changing and behavioral context in the Amazonian dwarf cichlid *Apistogramma hippolytae* (Perciformes). Neotrop. Ichthyol. 7:641–646.

[evl324-bib-0049] Rowland, W. J. 1999 Studying visual cues in fish behavior: a review of ethological techniques. Environ. Biol. Fishes 56:285–305.

[evl324-bib-0050] Salzburger, W. 2009 The interaction of sexually and naturally selected traits in the adaptive radiations of cichlid fishes. Mol. Ecol. 18:169–185. 18992003 10.1111/j.1365-294X.2008.03981.x

[evl324-bib-0051] Searcy, W. A. , and S. Nowicki . 2005 The evolution of animal communication: reliability and deception in signaling systems. Princeton Univ. Press, Princeton.

[evl324-bib-0052] Spinks, R. K. , M. Muschick , W. Salzburger , and H. F. Gante . 2017 Singing above the chorus: cooperative Princess cichlid fish (*Neolamprologus pulcher*) has high pitch. Hydrobiologia 791:115–125.

[evl324-bib-0053] Számadó, S. 2011a Long‐term commitment promotes honest status signalling. Anim. Behav. 82:295–302.

[evl324-bib-0054] Számadó, S. 2011b The cost of honesty and the fallacy of the handicap principle. Anim. Behav. 81:3–10.

[evl324-bib-0055] Számadó, S. 2012 The rise and fall of handicap principle: a commentary on the “Modelling and the fall and rise of the handicap principle.” Biol. Philos. 27:279–286.

[evl324-bib-0056] Taborsky, B. , and R. F. Oliveira . 2012 Social competence: an evolutionary approach. Trends Ecol. Evol. 27:679–688. 23040461 10.1016/j.tree.2012.09.003

[evl324-bib-0057] Tanaka, Y. 1996 Social selection and the evolution of animal signals. Evolution. 50:512–523. 28568955 10.1111/j.1558-5646.1996.tb03864.x

[evl324-bib-0058] Tibbetts, E. A. , and J. Dale . 2004 A socially enforced signal of quality in a paper wasp. Nature 432:218–222. 15538369 10.1038/nature02949

[evl324-bib-0059] Tibbetts, E. A. , and A. Izzo . 2010 Social punishment of dishonest signalers caused by mismatch between signal and behavior. Curr. Biol. 20:1637–1640. 20727756 10.1016/j.cub.2010.07.042

[evl324-bib-0060] Le Vin, A. L. , B. K. Mable , and K. E. Arnold . 2010 Kin recognition via phenotype matching in a cooperatively breeding cichlid, Neolamprologus pulcher. Anim. Behav. 79:1109–1114.

[evl324-bib-0061] Vorobyev, M. , R. Brandt , D. Peitsch , S. B. Laughlin , and R. Menzel . 2001 Colour thresholds and receptor noise: behaviour and physiology compared. Vision Res. 41:639–653. 11226508 10.1016/s0042-6989(00)00288-1

[evl324-bib-0062] Vorobyev, M. , and D. Osorio . 1998 Receptor noise as a determinant of colour thresholds. Proc. Biol. Sci. 265:351–358. 9523436 10.1098/rspb.1998.0302 PMC1688899

[evl324-bib-0063] Wagner, C. E. , L. J. Harmon , and O. Seehausen . 2012 Ecological opportunity and sexual selection together predict adaptive radiation. Nature 487:366–369. 22722840 10.1038/nature11144

[evl324-bib-0064] West‐Eberhard, M. J. 1983 Sexual selection, social competition, and speciation. Q. Rev. Biol. 58:155–183.

[evl324-bib-0065] Wong, M. , and S. Balshine . 2011 The evolution of cooperative breeding in the African cichlid fish, *Neolamprologus pulcher* . Biol. Rev. Camb. Philos. Soc. 86:511–530. 20849492 10.1111/j.1469-185X.2010.00158.x

[evl324-bib-0066] Zahavi, A. 1975 Mate selection—a selection for a handicap. J. Theor. Biol. 53:205–214. 1195756 10.1016/0022-5193(75)90111-3

[evl324-bib-0067] Zöttl, M. , D. Heg , N. Chervet , and M. Taborsky . 2013 Kinship reduces alloparental care in cooperative cichlids where helpers pay‐to‐stay. Nat. Commun. 4:1341. 23299891 10.1038/ncomms2344

