# Supplementary material for: Real‐time social selection maintains honesty of a dynamic visual signal in cooperative fish
Source: Evol Lett. 2017 Oct 25;1(5):269–78. doi: 10.1002/evl3.24 (PMC6121853; doi:10.1002/evl3.24)
Supplement: Supplementary file 1 — Figure S1. Data on the visual system of Neolamprologus brichardi and its light environment used to build visual models. Figure S2. Data on the light environment of Neolamprologus brichardi used to build visual models. Figure S3. Schematic representation of resource contest setup. Figure S4. Out‐of‐equilibrium manipulation of the horizontal facial stripe along axis of normal trait variation. Figure S5. Schematic representation of standard mirror image experimental setup. Figure S6. Spectral properties of dominant and subordinate individuals. Figure S7. Maxwell color triangles of dominant and subordinate individuals. Figure S8. Resource contest experiment. Figure S9. Aspect of subordinate individual. Figure S10. Spectral properties of dominant and subordinate individuals and impacts of paling on contrasts. Figure S11. Aggression level and latency to aggression induced by out‐of‐equilibrium manipulations of the horizontal facial stripe. Table S1. Ethogram of behavior repertoire of Neolamprologus brichardi Table S2. Cluster analysis of principal components of spectral data [file EVL3-1-269-s001.pdf]

SUPPLEMENTARY FILE FOR:

Real-time social selection maintains honesty of a dynamic visual signal in cooperative fish

Judith C. Bachmann,[1,2] Fabio Cortesi,[1,3] Matthew D. Hall,[4] N. Justin Marshall,[3] Walter Salzburger,[1] and Hugo F. Gante[1]

[1]Zoological Institute, Vesalgasse 1, University of Basel, 4051 Basel, Switzerland. [2]Current address: Department of Evolutionary Biology and Environmental Studies, University of Zurich, 8057 Zurich, Switzerland. [3]Queensland Brain Institute, University of Queensland, Brisbane Queensland 4072, Australia. [4]School of Biological Sciences, Monash University, Melbourne 3800, Australia. Correspondence: hugo.gante@unibas.ch

SUPPLEMENTARY METHODS:

Choice of species. The Princess of Burundi, *Neolamprologus brichardi* (Teleostei: Cichlidae), is a small (up to 8 cm in standard length) fish native to Lake Tanganyika, eastern Africa. Although *N. brichardi* has been synonymized with *N. pulcher* based on non-reciprocally monophyletic mitochondrial lineages (Duftner *et al.* 2007), genome-wide nuclear data suggest that hybridization and introgression explain these patterns and indicate that they are indeed good species (Gante *et al.* 2016). The two species differ in their facial pigmentation patterns, which are the focus of our study. The facial pigmentation of *N. brichardi* is very conspicuous to the human eye and consists of two black melanic stripes, arranged in a horizontal T-shape, surrounded by structural blue coloration, yellow pigmentation elements and a white branchiostegal membrane. Another less conspicuous stripe is present in the pre-orbital (lachrymal) area. The species has a beige body with fine orange elements in the posterior half and white-fringed fins (see Figure 2).

Princess cichlids perform the complete range of behaviors observed in the wild under lab conditions, which makes it an optimal species for behavioral studies in captivity (Balshine-Earn & Lotem 1998). Social groups can be found on coastal rocky substrates of Lake Tanganyika, usually between 3–50 m deep. The rocky substrate provides a territory with shelters and breeding grounds where adhesive eggs are spawned (Taborsky 1984; Heg *et al.* 2004). The breeding male is always the largest individual of the group, usually followed by the breeding female and subordinate helpers are the smallest (Wong & Balshine 2011). Groups aggressively defend their territory and dominant females have dominance behavior similar to dominant males, show high testosterone levels and brain arginine vasotocin expression (a neuropeptide involved in

vertebrate territorial, reproductive and social behaviors) (Aubin-Horth *et al.* 2007). Life history and behavioral traits create conditions for repeated interactions among individuals. Most of them involve submissive behaviors, followed by aggressive behaviors and only then territory maintenance (such as digging) and broodcare (Taborsky & Grantner 1998).

Study animals. *Neolamprologus brichardi* were raised in family groups and kept under controlled captive conditions at the Zoological Institute, University of Basel, Switzerland, on a 12:12 h light:dark regime, in tanks with about 1.5 cm of sand, a foam filter, a heater and terracotta flowerpots as shelters. Fish were fed a combination of newly hatched *Artemia* nauplii, commercial flakes and frozen cichlid food once or twice a day.

Color reflectance spectra. We measured spectral reflectance of *N. brichardi* facial patterns using a USB4000 spectrophotometer (Ocean Optics Inc.) and DH-2000-DUV Mikropack deuterium-halogen light source, connected to a laptop computer running Ocean Optics SpectraSuite software. Twenty individuals (10 females and 10 males) were anesthetized using a solution of KOI MED® Sleep (KOI&BONSAI, 0.5% v/v 2-Phenoxyethanol) before being transferred to a shallow tray filled with sufficient water to fully cover the fish and the probe. Because anesthetizing the fish before measuring their spectral reflectance may induce a short term darkening of their skin pigmentation we took care to measure reflectance after original conditions were re-established (~15 seconds). We measured spectral reflectance of various facial color patches with a 200 μm bifurcated optic UV/visible fiber, holding the bare end of the fiber at a 45° angle to prevent specular reflectance, and calibrating the percentage of light reflected at each wavelength from 350–750 nm with a Spectralon 99% white reflectance standard

(measurements always taken by the same person). We took at least ten measurements per facial pattern per individual that we subsequently averaged.

Visual system. To characterize the visual system of *N. brichardi*, we used published quantitative opsin data (Brawand *et al.* 2014; Schulte *et al.* 2014) and amino acid sequences from eye RNAseq data (Brawand *et al.* 2014) done on our stock of *N. brichardi*, and collected new ocular transmission measurements from wild specimens. Our *N. brichardi* express the UV-sensitive *SWS1*, and the two green-sensitive *RH2A* and *RH2B* opsin genes, which is a common opsin expression palette in cichlid species, including the ones from Lake Malawi (Figure S1A) (Hofmann *et al.* 2010). On comparisons of amino acid sequences of these three genes to the sequences of their Lake Malawi relatives we found that there are only minor differences between species. In particular, *N. brichardi* and *Metriaclima* (*Maylandia*) *zebra* show amino acid similarity of 95.4% (± 1.1%, SEM) at *SWS1*, 98% (± 0.7%, SEM) at *RH2Aα* and 97.7% (± 0.8%, SEM) at *RH2B* (similarities calculated in MEGA6) (Tamura *et al.* 2013). We measured ocular media transmission of the whole eye (by cutting a window in the back), cornea, and the lens from wild caught *N. brichardi* (Cape Kachese, Zambia, Lake Tanganyika; n = 3) to gain an understanding of the physical light filtering properties of the eye, following previously established protocols (Siebeck & Marshall 2001; Hofmann *et al.* 2010). We directed light from a pulsed xenon light source (Jaz-PX, Ocean Optics Inc.) through the pinhole using a 400 μm optical fiber and the ocular media and collected by a 100 μm optical fiber attached to a Jaz spectrometer (Ocean Optics Inc.), using a Spectralon 99% white standard as a reflection standard. We took at least three measurements per medium that we subsequently averaged, normalized spectra using their maximum transmission, and the wavelength at which 50%

transmission (T50) was reached was determined within the 300–750 nm interval (Siebeck & Marshall 2001; Hofmann *et al.* 2010).

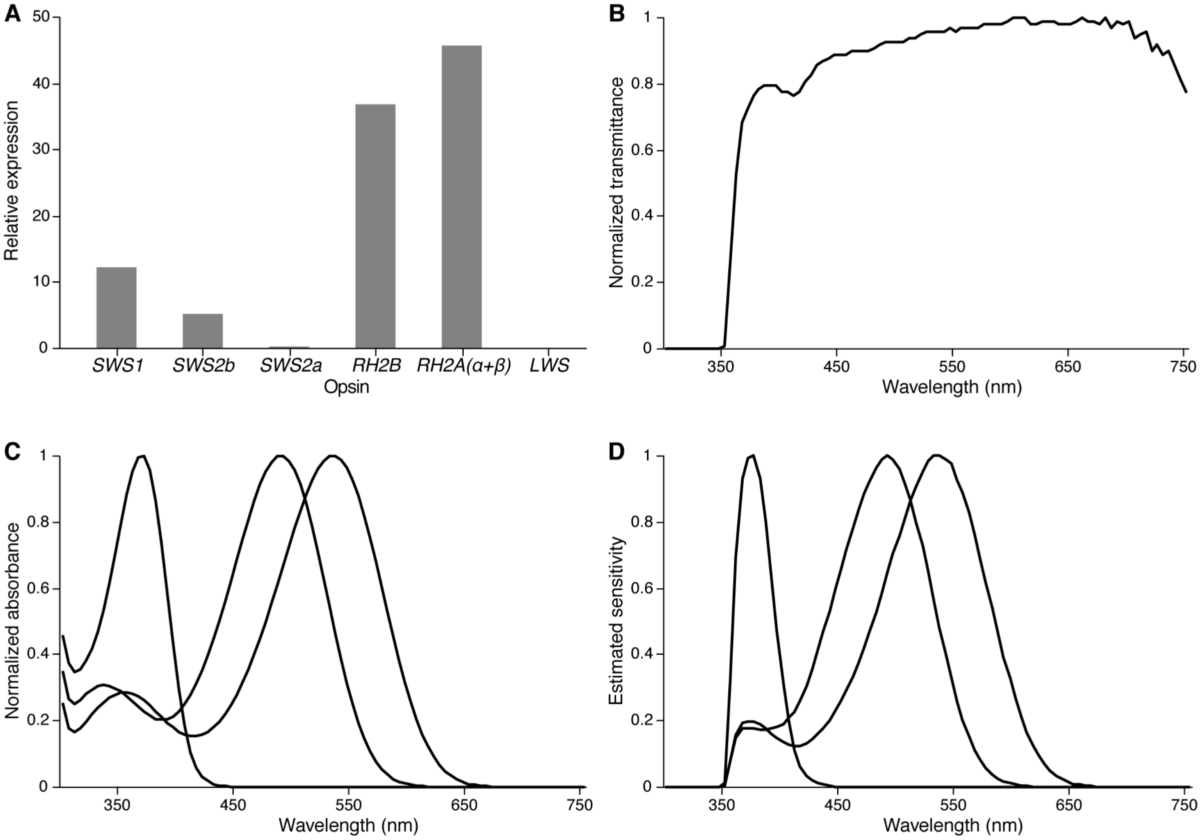

Figure S1. Data on the visual system of *Neolamprologus brichardi* and its light environment used to build visual models. A) Relative opsin expression of *N. brichardi* determined by RNAseq. B) Normalized transmittance of *N. brichardi* crystalline lens. C) Normalized absorbance of *SWS1*, *RH2B* and *RH2A*. D) Estimated visual sensitivities of *N. brichardi* incorporating the crystalline lens filtering medium.

We found the lens to be the limiting light transmission medium of the *N. brichardi* eye with a T50 cut-off value of 359 nm (Figure S1B). Based on our molecular assessment we followed Dalton *et al*. (Dalton *et al.* 2010), and used opsin absorbance spectra (λ_{max} = 368 nm for short wavelength (SWS), λ_{max} = 488 nm for mid wavelength (MWS), λ_{max} = 533 nm for long wavelength (LWS) (Levine & MacNichol 1979; Carleton *et al.* 2000) from *M.* (*M.*) *zebra*, a rock-dwelling cichlid species from lake Malawi, to reconstruct the visual sensitivities of *N.*

brichardi (Figure S1C). We then incorporated our lens transmission measurements to create a template of the visual system of *N. brichardi* to model how *N. brichardi* perceives color (Figure S1D).

Underwater light environment in Lake Tanganyika. We took measurements of the natural ambient light under which *N. brichardi* facial color patterns evolved, at Isanga Bay (Zambia, Lake Tanganyika) in September 2011 at depths of 3 m and 7 m (Figure S2). We measured illumination using a USB2000 spectrometer attached to a PALM-SPEC computer running native Ocean Optics software, enclosed in an underwater housing (Wills Camera Housings, Victoria, Australia). We used a shortened (60 cm) 1000 µm UV/visible optical-fiber with a cosine corrector to provide an 180º hemisphere to measure both, downwelling (by pointing the fiber upwards) and sidewelling light (pointing the fiber horizontally into the middle or towards the shore of the lake). However, there was no substantial difference in our overall conclusion when using either of the measurements.

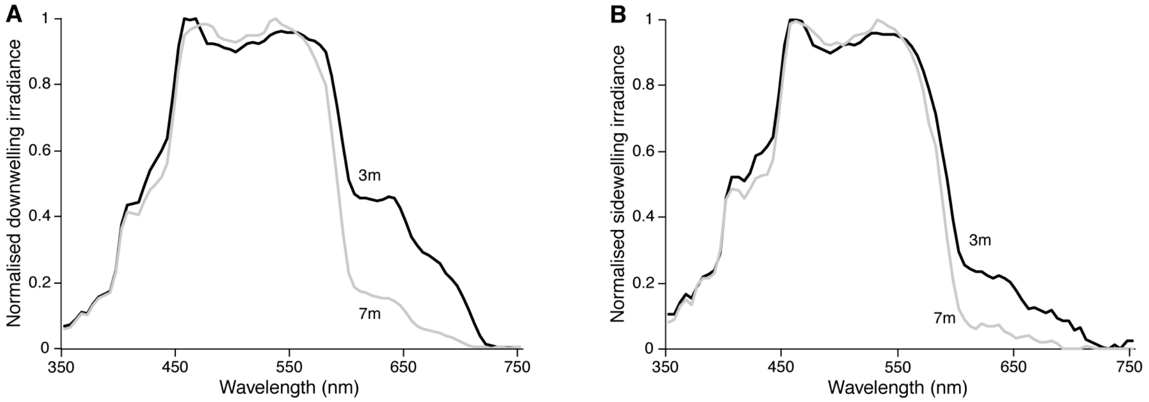

Figure S2. Data on the light environment of *Neolamprologus brichardi* used to build visual models. A) Normalized downwelling and B) sidewelling irradiance at two depths. Increasing water depth differentially reduces available longer light wavelengths.

Visual modeling to determine signal design properties. We used a theoretical visual model (Vorobyev & Osorio 1998; Vorobyev *et al.* 2001) to quantify the chromatic and achromatic contrasts between the facial pattern elements using the *N. brichardi* visual system and assuming ambient light conditions as measured from their natural habitat (see above). The chromatic model calculates the color distance (ΔS) within the visual space of the fish, where low values of ΔS denote similar colors and high values of ΔS indicate chromatically different colors. When calculating chromatic distances between color patches, luminosity is disregarded within the model, the colors are assumed to be encoded by an opponency mechanism based on the sensitivities of the fish visual system, and chromatic discrimination is thought to be limited by photoreceptor noise determined by the relative proportion of each photoreceptor (Vorobyev & Osorio 1998; Vorobyev *et al.* 2001). The receptor quantum catch (q_i) in the photoreceptor cell of type i is calculated as

$$q_i = \int Ri(\lambda)S(\lambda)I(\lambda)d\lambda$$

where λ denotes the wavelength, $Ri(\lambda)$ the spectral sensitivity of the photoreceptor cell, $S(\lambda)$ the spectral reflectance of the color patch, $I(\lambda)$ the illumination spectrum entering the eye and integration is over the range of 350–750 nm (equation 1 of Vorobyev & Osorio (Vorobyev & Osorio 1998)). Illumination was set as measured at a depth of 7 m and coming from above (no difference was found when using spot tests and illumination at 3 m). In the absence of retinal morphological and physiological data for our study species, we based the relative proportion of cone receptors on studies from other cichlid fishes, which frequently have a square pattern (Fernald 1981; van der Meer & Bowmaker 1995; Dalton *et al.* 2014) with a ratio of 1:2:2 (SWS:MWS:LWS), which is consistent with RNAseq data for *N. brichardi* (Figure S1A). The weber fraction (ω) was set to assume a 0.05 LWS noise threshold, which is a conservative

approach representing approximately half the sensitivity of the human LWS cone system (Wyszecki & Stiles. W. S. 2000). We predict that the more ΔS increases above the threshold of 1 JND (just noticeable difference) (Vorobyev & Osorio 1998; Endler 2012), the more distinguishable colors become from one another, which might be especially important for long-range signals where intervening water and particles start to blur colors (Marshall *et al.* 2003).

In addition to chromatic contrasts we also calculated achromatic contrasts as a second property of the visual signal. Long wavelength receptors are thought to be responsible when perceiving differences in luminance (for discussion see (Marshall *et al.* 2003)) and we therefore used the differences in the natural logarithm quantum catch (Q) of the long wavelength receptor (L) to calculate luminance differences between color patches using

$$\Delta L = ln(Q_{LpatchX}) - ln(Q_{LpatchY}).$$

We note that conspicuousness depends on distance between sender and receiver. Because even "bright" colors can blend at a distance depending on their spatial frequency, and allow camouflaging against appropriate backgrounds (Marshall 2000; Barnett *et al.* 2017), we assume that sender and receiver are at close range (Figure 1), which allows effective signaling.

Resource contest experiment to determine signal message. A total of 40 *N. brichardi* (20 males and 20 females) originating from several stock tanks were sexed (by examination of the genital papilla), measured (standard length [SL], taken as the distance between the tip of the snout and the insertion of the middle caudal fin rays) and weighed (body mass [BM], taken after one day fasting). Female SL was 5.41 ± 0.55 cm (mean ± standard deviation) and BM was 4.30 ± 1.34 g. Male SL was 5.62 ± 0.56 cm and BM 4.67 ± 1.48 g. To control for daily variation in behaviors, all territorial dyadic combats were always conducted between 11AM and 1PM

(Oliveira *et al.* 2001). Combats were performed in an aquarium (60 × 30 × 30 cm) divided in the middle of the longer side into two equal compartments by a removable opaque grey plastic barrier (Figure S3). The conditions in both compartments were the same: each had a filter, a heater, ca. 2 cm of sand and a quarter of a terracotta flowerpot (12 cm in diameter, 10 cm long) adjacent to the barrier. Due to the social nature of these fish, small opposite-sexed conspecifics (one per compartment) were introduced in transparent plastic bottles to encourage territory establishment of the focal fish. Test fish were caught from their tanks of origin and released in one of the two compartments to establish their territory in the quarter flowerpot for three days. The compartment was randomly selected. Fish were fed commercial flakes or frozen cichlid food twice a day and at least one hour before starting the trial to control for effects of feeding regime on behavior (Fisher & Rosenthal 2006). Following this acclimation period the visual barrier was removed, merging the territories of the two fish, and merging the flowerpot shelter into one. This procedure guarantees that both fish have simultaneous ownership over a territory and that they cannot divide this resource after the barrier has been removed. *Neolamprologus brichardi* is a highly territorial species and immediately starts to combat for ownership of the shelter (Taves *et al.* 2009; Reddon *et al.* 2011). To avoid disturbance by the experimenter, the interactions of the fish were videotaped with a Sony HDR XR 550VE camcorder. After each trial ended, fish were moved to separate holding nets in their original tanks.

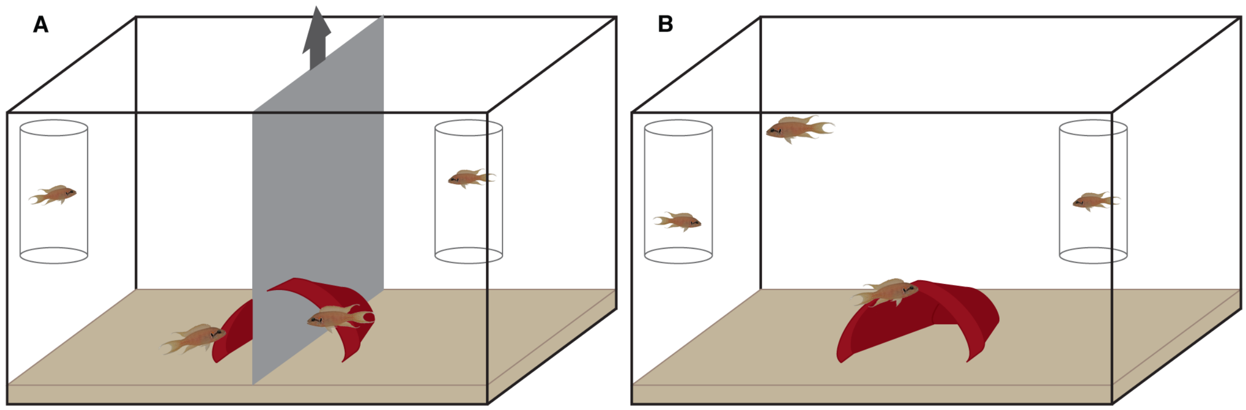

Figure S3. Schematic representation of resource contest setup. A) fish are visually separated and allowed to establish their territory in the terracotta flowerpot for three days, after which the opaque divider is removed and territories/shelters merged (B) and individuals are allowed to fight over non-divisible territorial resource for 20 minutes. Observer could see the fish without being seen to control for dangerous levels of aggression.

The winner was declared as the fish from which the loser fled three times without counter-strike or constantly held a submissive posture (Reddon *et al.* 2011; Dijkstra *et al.* 2012). Alternatively, a fish was declared winner if it owned the flowerpot at the end of the combat (i.e. the most valuable resource of the territory). Behaviors of both fish were counted and fit into four categories of recorded behaviors due to their very diverse behavioral repertoire (Table S1), which is consistent with other studies of *Neolamprologus* (Balshine *et al.* 2001; Sopinka *et al.* 2009; Taves *et al.* 2009). We recorded the intensity of the horizontal facial stripe (pale or dark) in both fish at the beginning and at the end of experiments.

Facial stripe out-of-equilibrium manipulation and standard mirror image stimulation experiment to determine sources of communication reliability. Standard mirror image stimulation (MIS) experiments were used to determine if *N. brichardi* are able to recognize and punish unreliable signaling by measuring the response of each individual to its own mirror image.

Table S1. Ethogram of behavior repertoire of *Neolamprologus brichardi*

Category	Behaviors	Description
**Contact aggression**	Bite	Focal fish bites another fish
	Chase	Focal fish follows another fish
	Displace	Focal fish swims towards another fish, forcing it to move
	Mouth-lock*	Two fish lock jaws and push against each other
	Ram	Focal fish hits another fish with its head, but jaws remain closed
**Display aggression**	Aggressive posture	Focal fish lowers its head towards another fish and shows the side of its body with spread fins
	Head shake	Fish tosses its head from left to right
	Puffed throat	Fish opens operculum and lower jaw cavity
**Submission**	Bitten	Focal fish gets bitten by another fish
	Flee	Focal fish swims away from another fish
	Submissive posture	Focal fish has a (nearly) vertical position, with the head directing upwards
	Submissive display	Focal fish is positioned with a submissive posture accompanied by a quivering caudal fin
**Territoriality**	Body digging	Focal fish quivers its body on the substrate and moves sand
	Digging	Focal fish takes sand in its mouth, sometimes swims to a different area and spits it out
	Lookout	Focal fish observes another fish from its shelter
	Hover	Focal fish defends brood chamber, inhibit other fish from entering
	Cleaning	Focal fish removes algae from shelter by nibbling on them

* both fish get the score

A total of 49 *N. brichardi* (25 males and 24 females) originating from several stock tanks were sexed, measured (SL), and weighed (BM) as in the resource contest experiment (above). Female SL was 5.90 ± 0.74 cm (mean ± standard deviation) and BM was 5.19 ± 2.21 g. Male SL was 5.92 ± 0.84 cm and BM 5.20 ± 2.62 g. Before fish were tested, they were separated from their social group for two days and kept in a pre-test tank (40 × 25 × 25 cm), covered on all four sides to minimize disturbance. This tank contained a flowerpot arch placed adjacent to one wall so fish learned to use this as a shelter instead of a closed flowerpot.

After this isolation period of two days, during which they became territorial, fish were gently netted out of the pre-test tank, partially anesthetized with KOI MED® Sleep (KOI&BONSAI, 0.5% v/v 2-Phenoxyethanol) and the horizontal facial stripe was manipulated randomly in one of three different ways. To control for individual effects of aggression levels between behavioral types (Bell 2007), fish were tested twice with two different treatments. Order of treatment was randomized:

1. Darkened facial stripe: The facial stripe was brushed with black waterproof liquid eyeliner (Collection 2000, USA). To control for the paling treatment (see below), wound snow and wound spray (KOI MED®) were applied in the head region above the facial stripes.

2. Paled facial stripe: To control for the darkening treatment, the facial stripe was first painted with the black waterproof liquid eyeliner (Collection 2000, USA) and then covered up with 'Wound Snow' and 'Wound Spray' (KOI MED®) to produce a paling effect.

3. Control sham-manipulation: Same treatment as 2. applied in the head region above the facial stripe as in 1., so the facial stripe was left un-manipulated.

Sham-manipulating (3) and controlling for the opposite treatment (1 and 2) help to homogenize handling time of each fish, and guarantee that behaviors observed are not induced by exposure to a particular compound of the products used, but rather to the facial stripe manipulation. Spectral reflectance measurements show that both treatments resulted in the desired effect of darkening and paling along the axis of variation of, and slightly more extreme than, non-manipulated horizontal stripes. A principal components analysis (PCA) of spectral reflectance data clearly groups black 'eyeliner' with dark melanic stripes (indicative of dominant fish) and groups 'Wound Snow' with pale horizontal stripe, lachrymal stripe and head (indicative

of subordinate fish) (Figure S4). A cluster analysis conducted with R package mclust (Fraley *et al.* 2014) confirms this visual assessment (Table S2).

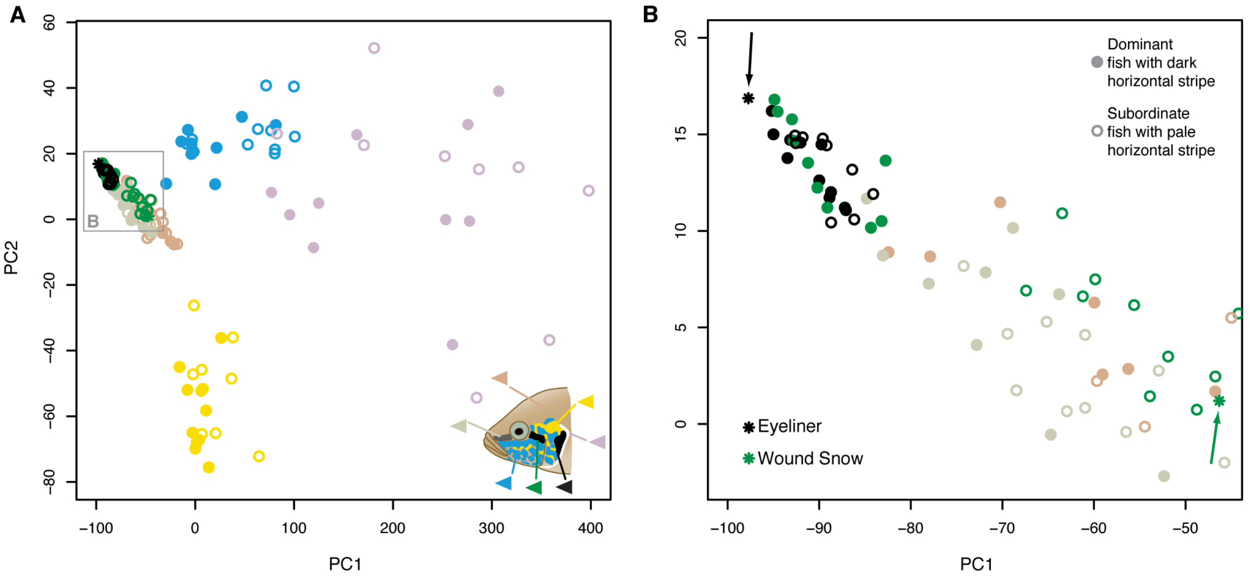

Figure S4. Out-of-equilibrium manipulation of the horizontal facial stripe along axis of normal trait variation. A) and B) Principal components analysis of spectral data and signal experimental manipulation. Principal Components 1 and 2 explain 96.5% of the variance (90.8% and 5.7%, respectively) and clearly separate different colors. B) Zoom in at the melanistic area of the plot shows that 'Eyeliner' is similar to dark melanic stripes of dominant fish, while 'Wound Snow' is similar to pale melanistic stripes of subordinates and in both cases create a more extreme out-of-equilibrium phenotype (arrows). See also Table S2 for clustering of Principal Components.

The test setup consisted of an aquarium (40 × 25 × 25 cm) with a 2.84 mm-thick plane glass mirror (25 × 25 cm), placed inside the tank, behind a terracotta flowerpot arch (10 cm in diameter; 3 cm wide) at a sidewall. Using a flowerpot arch instead of a closed flowerpot guaranteed that the fish could see their reflection at all times, including inside the shelter, avoiding the generation of impossible reflection angles that could confuse them. At the beginning the arch and the mirror were hidden behind an opaque grey plastic barrier. After removal of the opaque barrier, the mirror image should reflect a conspecific territory owner to the non-territorial test fish.

Table S2. Cluster analysis of principal components of spectral data

	Clusters				
Color patch	1	2	3	4	5
Eyeliner	0	1	0	0	0
Wound Snow	1	0	0	0	0
Vertical facial stripe (D)	0	10	0	0	0
Vertical facial stripe (S)	0	10	0	0	0
Horizontal facial stripe (D)	1	9	0	0	0
Horizontal facial stripe (S)	10	0	0	0	0
Lachrymal stripe (D)	8	2	0	0	0
Lachrymal stripe (S)	10	0	0	0	0
Head (D)	9	1	0	0	0
Head (S)	10	0	0	0	0
Blue (D)	0	0	10	0	0
Blue (S)	0	0	10	0	0
Branchiostegal (D)	0	0	0	0	10
Branchiostegal (S)	0	0	1	0	9
Yellow (D)	0	0	0	9	1
Yellow (S)	0	0	0	9	1

Five clusters were identified. ‘Eyeliner’ clusters together with black melanistic stripes (cluster 2), while ‘Wound Snow’ clusters with pale horizontal stripe, lachrymal stripe and head (cluster 1). D: dominant. S: subordinate. Refer to Figures 5 and S4.

This setup addresses common limitations faced when presenting manipulated individuals to focal dominant, territorial individuals (Bradbury & Vehrencamp 2011). In our setup, the focal fish act as intruders and test the repellent effect of the manipulated signals in individuals of the same size they perceive as territory owners (Figure S5). After manipulation of the facial stripe the fish were released into the test tank compartment without the flowerpot arch and mirror, and allowed to recover for 5 min from anesthesia and treatment. For motivational purposes and to ease acclimation to these new surroundings, fish were fed a little amount of frozen newly hatched *Artemia* nauplii. After the recovery period, the opaque barrier was removed and the individual fish could interact with their mirror image. To control for daily variation in behaviors,

all experiments were conducted between 9AM and 11:30AM (Oliveira *et al.* 2001). All aggressive (display and contact) and submissive behaviors towards the mirror image were counted during a period of 2.5 min from a video recording (Sony camcorder, see above), starting after the removal of the opaque barrier.

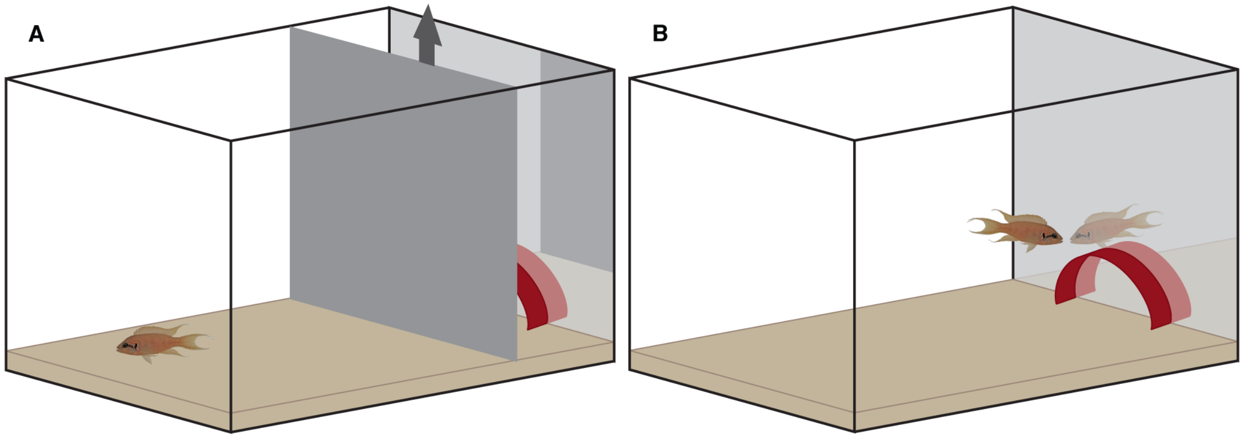

Figure S5. Schematic representation of standard mirror image experimental setup. A) fish with manipulated signals are introduced to a bare side of the tank with no shelters. After opaque divider is removed (B), individuals can see a shelter and a fish holding a territory next to it (i.e. their mirror image) and are allowed to interact with it.

SUPPLEMENTARY RESULTS AND DISCUSSION:

Spectral data. We assessed spectra of each individual facial color patches based on the wavelength at which light was reflected and the shape of the reflectance curves (Figures 3A, 3B, S6). Changes in horizontal stripe luminance create two discrete phenotypic states, dark and pale – reflecting two dominance levels, dominant and subordinate, respectively. Only horizontal facial stripe spectra do not overlap between dark and pale fish, other colors overlap to various degrees. This is supported by both perceptual (e.g. Figure 3) and non-perceptual methods (e.g. Figure S4).

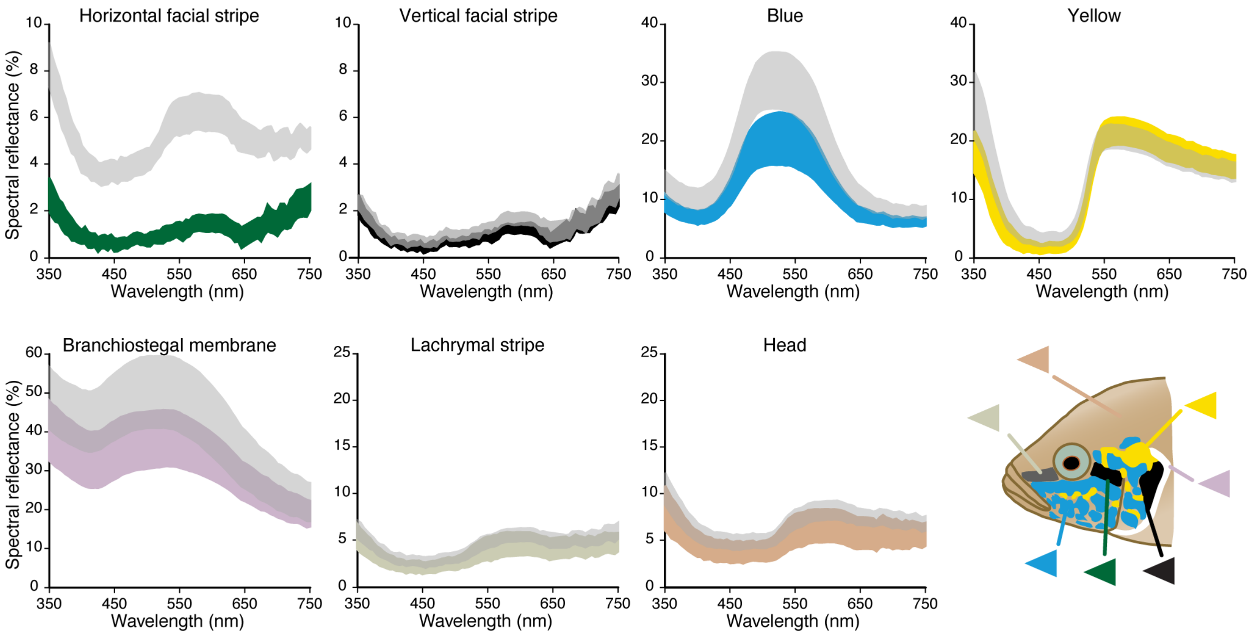

Figure S6. Spectral properties of dominant and subordinate individuals. 95% confidence intervals of spectral reflectance in dominant and subordinate individuals. Colored intervals refer to color patches in individuals with dark horizontal stripe (dominant), while light grey intervals refer to equivalent patches in individuals with pale horizontal stripe (subordinates).

In addition, using Maxwell color triangles to represent the trichromatic visual space of *N. brichardi*, we confirm that all color patches except for the horizontal facial stripe (Figure S7, top

left), overlap between dominant and subordinate fish (see (Dalton *et al.* 2010) for methodological details).

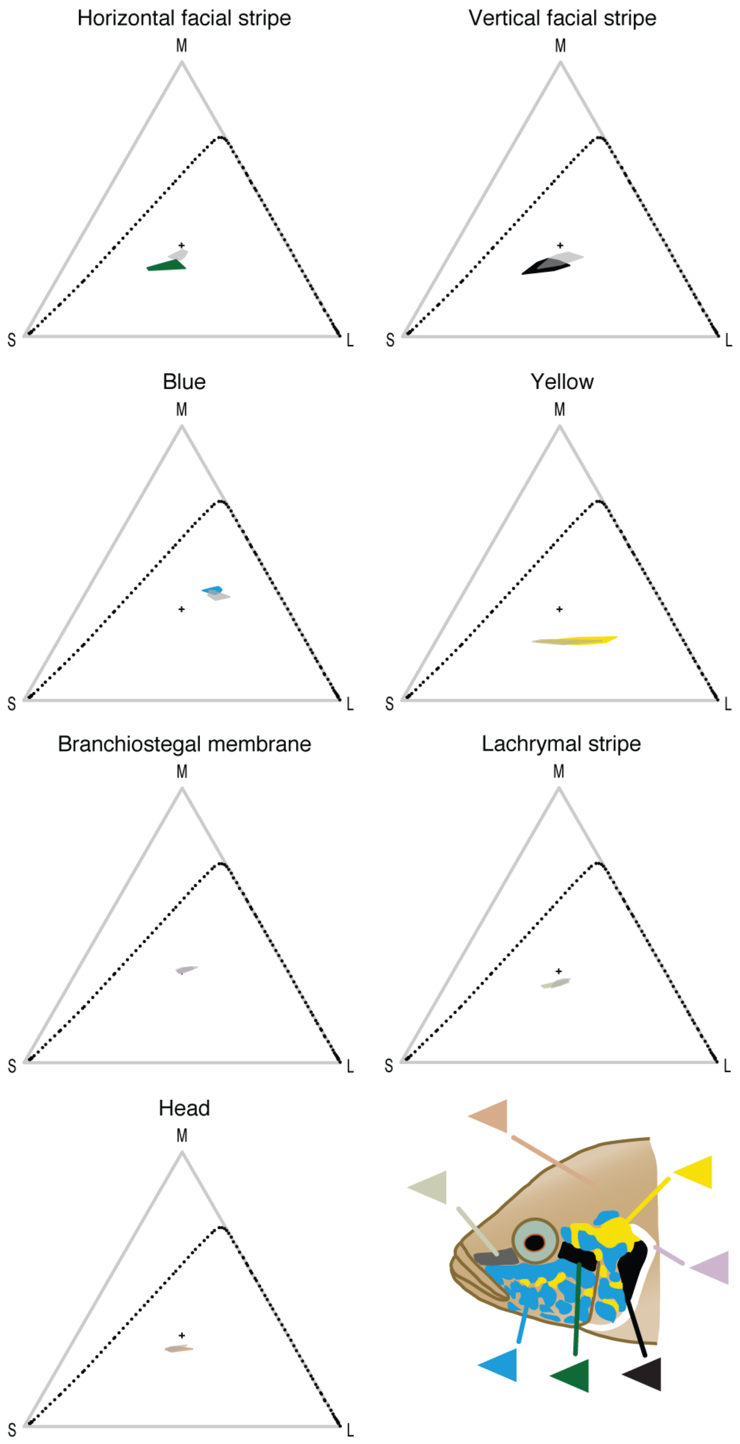

Figure S7. Maxwell color triangles of dominant and subordinate individuals. For each color patch colored polygons represent dominant individuals (with dark horizontal stripe) and grey polygons

represent subordinate individuals (with pale horizontal stripe). S, M, and L stand for short, mid and long wavelength photoreceptors. Stippled line represents a hypothetical monochromatic locus, calculated at 1nm intervals, which determines the area inside the triangle that can be occupied by colors seen by the fish, inferred from the degree of overlap in the absorbance spectra of photoreceptors. Pure white lies at the center of the triangle (cross).

Territorial combat. Larger and more aggressive individuals are more likely to win territorial disputes (Figure S8).

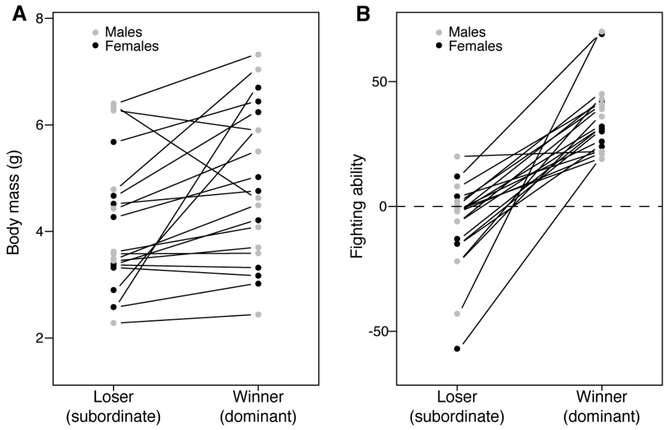

Figure S8. Resource contest experiment. A) and B) Winners of combats for territorial resource are larger and fight more aggressively than losers. Lines connect fish dyads.

Paling of the horizontal stripe. As the individual assumes a subordinate behavior, physiological color changes take place in a few seconds (Figure S9).

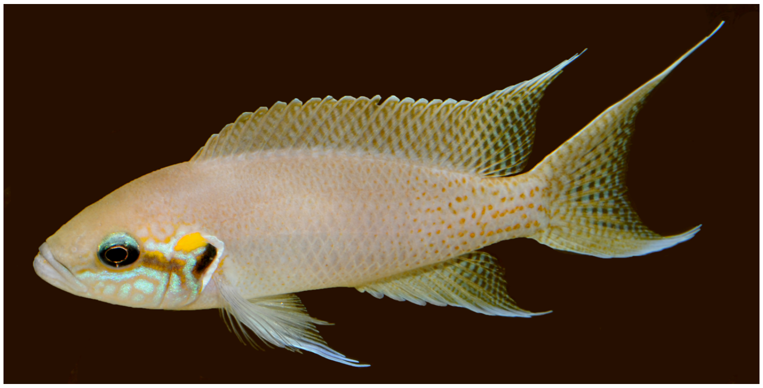

Figure S9. Aspect of subordinate individual. Note the distinctively pale horizontal stripe in contrast to dark horizontal stripe of the fish in Figure 2.

Theoretical visual models confirm that *chromatic* conspicuousness is unaffected even after paling takes place (Figure S10A, empty circles), while in the case of *achromatic* contrasts , adjacent contrasts become higher than non-adjacent contrasts in submissive fish (Figure S10B, empty circles).

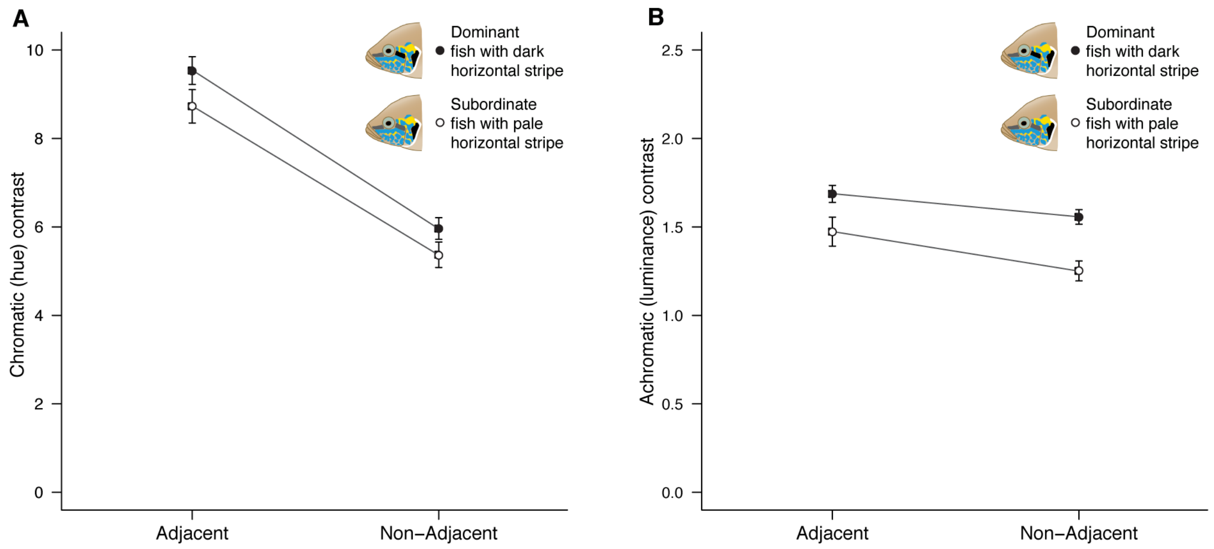

Figure S10. Spectral properties of dominant and subordinate individuals and impacts of paling on contrasts. A) Chromatic contrast (mean ± SEM) is achieved by adjacency of color elements and is not influenced by darkness of horizontal stripe. B) Achromatic contrast (mean ± SEM) is achieved by paling of the horizontal stripe, which has a differential influence on adjacent and non-adjacent color elements.

Detection of liars. Testing of each individual with two manipulations of the horizontal stripe allows detecting increased and faster aggression toward unreliable signaling, as indicated by the reaction norms (Figure S11). While the precise mechanism that allow receivers to detect cheating is not yet known and deserves attention in itself, it is possible that like paper wasps (Tibbetts & Izzo 2010), fish have the ability to detect a mismatch between color signals and associated behaviors to assess the information content of signals. For instance, dominant Princess of Burundi cichlids spread their fins and tilt their bodies upwards while displaying, while individuals trying to appease an opponent assume an antithetical behavior (see Figure S9).

Interestingly, social selection against cheaters in *N. brichardi* is not symmetrical, which supports the view that signaling systems are more likely disrupted by 'Trojans' (signaling weakness) than by 'bluffers' (signaling strength) (Számadó 2011).

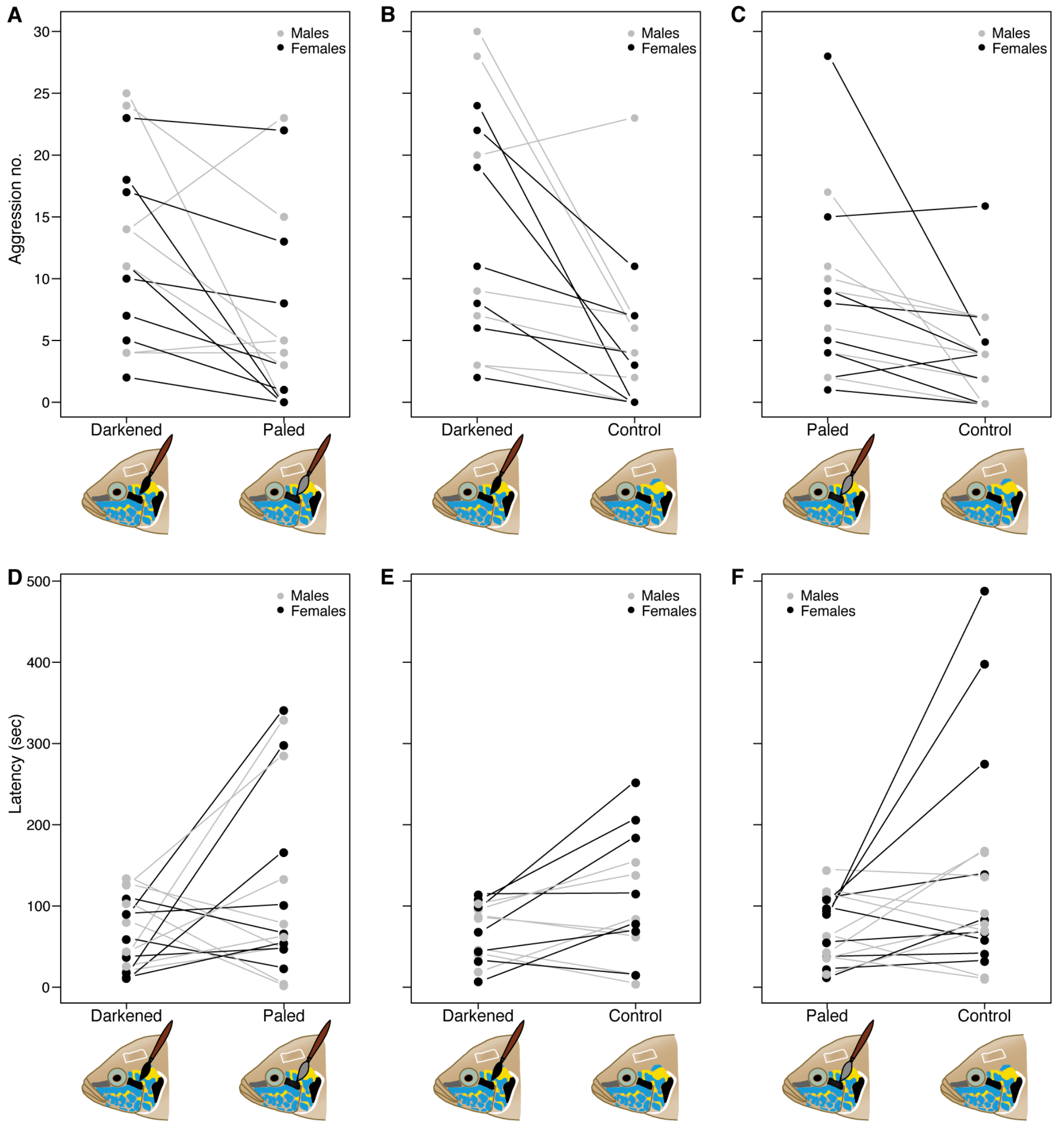

Figure S11. Aggression level and latency to aggression induced by out-of-equilibrium manipulations of the horizontal facial stripe. A), B) and C) Reaction norms of amount of aggression incurred by individuals with out-of-equilibrium signals and controls. D), E) and F) Reaction norms of latency to aggression in the same treatments. Retaliation costs are highest and fastest for individuals with artificially darkened stripe ('bluffers', i.e. darkened), followed by individuals with paled stripe ('Trojans', i.e. paled), and then controls (i.e. reliable signalers), which were the ones that received the lowest amount of aggression, at a later stage of the stripe manipulation experiment. Lines connect the same individual, tested twice with two different treatments. Refer to Figure 5.

Detection and punishment of weak individuals that signal strength ('bluffers') by receivers might be favored by selection more than punishment of strong individuals that do not signal reliably ('Trojans') because of possible fitness consequences of fighting a strong individual. It is thus possible that in some scenarios 'Trojan' detection by receivers does not evolve at all (Owens & Hartley 1991), or that statistical detection of punishment requires larger sample sizes, for a comparatively smaller level of punishment.

REFERENCES

Aubin-Horth N, Desjardins JK, Martei YM, Balshine S, Hofmann HA (2007) Masculinized dominant females in a cooperatively breeding species. *Molecular Ecology*, **16**, 1349–1358.

Balshine S, Leach B, Neat F *et al.* (2001) Correlates of group size in a cooperatively breeding cichlid fish (*Neolamprologus pulcher*). *Behavioral Ecology and Sociobiology*, **50**, 134–140.

Balshine-Earn S, Lotem A (1998) Individual recognition in a cooperatively breeding cichlid: evidence from video playback experiments. *Behaviour*, **135**, 369–386.

Barnett JB, Cuthill IC, Scott-Samuel NE (2017) Distance-dependent pattern blending can camouflage salient aposematic signals. *Proceedings of the Royal Society B: Biological Sciences*, **284**, 20170128.

Bell AM (2007) Future directions in behavioural syndromes research. *Proceedings of the Royal Society B: Biological Sciences*, **274**, 755–761.

Bradbury JW, Vehrencamp SL (2011) *Principles of Animal Communication, Second Edition*. Sinauer Associates, Inc.; 2 edition.

Brawand D, Wagner CE, Li YI *et al.* (2014) The genomic substrate for adaptive radiation in African cichlid fish. *Nature*, **513**, 375–381.

Carleton KL, Ha FI, Kocher TD (2000) Visual pigments of African cichlid fishes: evidence for ultraviolet vision from microspectrophotometry and DNA sequences. *Vision Research*, **40**, 879–890.

Dalton BE, Cronin TW, Marshall NJ, Carleton KL (2010) The fish eye view: are cichlids conspicuous? *The Journal of Experimental Biology*, **213**, 2243–2255.

Dalton BE, Loew ER, Cronin TW, Carleton KL (2014) Spectral tuning by opsin coexpression in retinal regions that view different parts of the visual field. *Proceedings of the Royal Society B: Biological Sciences*, **281**.

Dijkstra PD, Schaafsma SM, Hofmann HA, Groothuis TGG (2012) "Winner effect" without winning: unresolved social conflicts increase the probability of winning a subsequent contest in a cichlid fish. *Physiology & Behavior*, **105**, 489–492.

Duftner N, Sefc KM, Koblmüller S *et al.* (2007) Parallel evolution of facial stripe patterns in the *Neolamprologus brichardi/pulcher* species complex endemic to Lake Tanganyika. *Molecular Phylogenetics and Evolution*, **45**, 706–715.

Endler JA (2012) A framework for analysing colour pattern geometry: adjacent colours. *Biological Journal of the Linnean Society*, **107**, 233–253.

Fernald RD (1981) Chromatic organization of a cichlid fish retina. *Vision Research*, **21**, 1749–1753.

Fisher HS, Rosenthal GG (2006) Hungry females show stronger mating preferences. *Behavioral Ecology*, **17**, 979–981.

Fraley C, Raftery A, Scrucca L (2014) mclust: Normal Mixture Modeling for Model-Based Clustering, Classification, and Density Estimation.

Gante HF, Matschiner M, Malmstrøm M *et al.* (2016) Genomics of speciation and introgression in Princess cichlid fishes from Lake Tanganyika. *Molecular Ecology*, **25**, 6143–6161.

Heg D, Bachar Z, Brouwer L, Taborsky M (2004) Predation risk is an ecological constraint for helper dispersal in a cooperatively breeding cichlid. *Proceedings. Biological sciences / The Royal Society*, **271**, 2367–2374.

Hofmann CM, O'Quin KE, Justin Marshall N, Carleton KL (2010) The relationship between lens transmission and opsin gene expression in cichlids from Lake Malawi. *Vision Research*, **50**, 357–363.

Levine JS, MacNichol EF (1979) Visual pigments in teleost fishes: effects of habitat, microhabitat, and behavior on visual system evolution. *Sensory Processes*, **3**, 95–131.

Marshall NJ (2000) Communication and camouflage with the same "bright" colours in reef fishes. *Philosophical Transactions of the Royal Society of London. Series B, Biological Sciences*, **355**, 1243–1248.

Marshall NJ, Jennings K, McFarland WN, Loew ER, Losey GS (2003) Visual biology of Hawaiian coral reef fishes. III. Environmental light and an integrated approach to the ecology of reef fish vision (WL Montgomery, Ed,). *Copeia*, **2003**, 467–480.

Van der Meer HJ, Bowmaker JK (1995) lnterspecific variation of photoreceptors in four co-existing haplochromine cichlid fishes. *Brain, Behavior and Evolution*, **45**, 232–240.

Oliveira RF, Lopes M, Carneiro LA, Canário AVM (2001) Watching fights raises fish hormone levels. *Nature*, **409**, 2001.

Owens IPF, Hartley IR (1991) “Trojan Sparrows”: evolutionary consequences of dishonest invasion for the badges-of-status model. *The American Naturalist*, **138**, 1187–1205.

Reddon AR, Voisin MR, Menon N *et al.* (2011) Rules of engagement for resource contests in a social fish. *Animal Behaviour*, **82**, 93–99.

Schulte JE, O’Brien CS, Conte MA, O’Quin KE, Carleton KL (2014) Interspecific variation in Rx1 expression controls opsin expression and causes visual system diversity in African cichlid fishes. *Molecular Biology and Evolution*, **31**, 2297–2308.

Siebeck UE, Marshall NJ (2001) Ocular media transmission of coral reef fish — can coral reef fish see ultraviolet light? *Vision Research*, **41**, 133–149.

Sopinka NM, Fitzpatrick JL, Desjardins JK *et al.* (2009) Liver size reveals social status in the African cichlid *Neolamprologus pulcher*. *Journal of Fish Biology*, **75**, 1–16.

Számadó S (2011) Long-term commitment promotes honest status signalling. *Animal Behaviour*, **82**, 295–302.

Taborsky M (1984) Broodcare helpers in the cichlid fish *Lamprologus brichardi*: their costs and benefits. *Animal Behaviour*, **32**, 1236–1252.

Taborsky M, Grantner A (1998) Behavioural time – energy budgets of cooperatively breeding *Neolamprologus pulcher* (Pisces : Cichlidae). *Animal Behaviour*, **56**, 1375–1382.

Tamura K, Stecher G, Peterson D, Filipski A, Kumar S (2013) MEGA6: Molecular Evolutionary Genetics Analysis. *Molecular Biology and Evolution*, **30**, 2725–2729.

Taves MD, Desjardins JK, Mishra S, Balshine S (2009) Androgens and dominance: sex-specific patterns in a highly social fish (Neolamprologus pulcher). *General and Comparative Endocrinology*, **161**, 202–207.

Tibbetts EA, Izzo A (2010) Social punishment of dishonest signalers caused by mismatch between signal and behavior. *Current Biology*, **20**, 1637–1640.

Vorobyev M, Brandt R, Peitsch D, Laughlin SB, Menzel R (2001) Colour thresholds and receptor noise: behaviour and physiology compared. *Vision Research*, **41**, 639–653.

Vorobyev M, Osorio D (1998) Receptor noise as a determinant of colour thresholds. *Proceedings. Biological sciences / The Royal Society*, **265**, 351–358.

Wong M, Balshine S (2011) The evolution of cooperative breeding in the African cichlid fish, *Neolamprologus pulcher*. *Biological Reviews of the Cambridge Philosophical Society*, **86**, 511–530.

Wyszecki G, Stiles. W. S. (2000) *Color science — concepts and methods, quantitative data and formulae*. Wiley, New York.
